# Switching Beyond Rituximab in Primary Glomerular Diseases: A Systematic Review of Within-Patient Efficacy and Safety of Non-Rituximab Anti-CD20 Antibodies

**DOI:** 10.3390/antib15050095

**Published:** 2026-10-10

**Authors:** Francesca K. Martino, Lucia F. Stefanelli, Leda Cattarin, Dorella Del Prete, Anna Basso, Federico Nalesso

**Affiliations:** Nephrology, Dialysis and Transplantation Unit, Department of Medicine (DIMED), University of Padova, 35128 Padua, Italy; luciafederica.stefanelli@unipd.it (L.F.S.); leda.cattarin@aopd.veneto.it (L.C.); dorella.delprete@unipd.it (D.D.P.); anna.basso@aopd.veneto.it (A.B.); federico.nalesso@unipd.it (F.N.)

**Keywords:** rituximab, anti-CD20 antibody, obinutuzumab, ofatumumab, glomerulonephritis, membranous nephropathy, minimal change disease, focal segmental glomerulosclerosis, IgA nephropathy, remission

## Abstract

**Background/Objectives:** Rituximab is widely used in primary glomerular diseases, but treatment may fail for several reasons, including primary non-response, incomplete response, relapse, intolerance or anti-rituximab antibodies. We assessed within-patient efficacy and safety after switching to another anti-CD20 antibody, considering the reason for switching. **Methods:** PubMed and Scopus were searched from inception to 15 May 2026 without language restrictions. Eligible reports included adults with primary glomerular disease treated with a non-rituximab anti-CD20 antibody after rituximab and reporting within-patient outcomes. Risk of bias was assessed with design-specific JBI tools and certainty with GRADE. Heterogeneity precluded meta-analysis, and findings were synthesized narratively. **Results:** A total of 309 patients were included across 43 studies: 283 had membranous nephropathy (MN), 18 had minimal change disease (MCD), 6 had focal segmental glomerulosclerosis (FSGS), and 2 had IgA nephropathy (IgAN). Obinutuzumab was the principal drug used, and the main reasons for switching were primary non-response, with immunization, relapse, or intolerance occurring less frequently. In MN, among the 262 patients with available response data, 217 achieved complete or partial remission, and relapse was rare. For MCD, 17 of 18 patients with active disease attained remission, and only one relapsed during follow-up. On the other hand, five out of the six FSGS patients responded, but three of them relapsed. The two patients with IgAN achieved either complete or partial remission. Notably, these results may not apply to conventional IgAN, since one biopsy showed contextually MCD and the other was proliferative and crescentic. Infusion-related reactions were the most frequent adverse events and were generally mild, while infections were the main issue during the follow-up. Evidence certainty for all outcomes was very low. **Conclusions:** Switching to a non-rituximab anti-CD20 antibody may be a feasible rescue strategy, most convincingly after intolerance, rituximab immunization, or relapse. The uncontrolled, very-low-certainty evidence precludes firm recommendations, and controlled studies are warranted.

## 1. Introduction

Anti-CD20 monoclonal antibodies have changed the treatment of several hematological malignancies and autoimmune diseases [1]. Their effects are not limited to reducing circulating antibodies; they also include the immunomodulation of B cells, which contribute to antigen presentation, cytokine production, co-stimulation, and regulation of T-cell responses. This broader immunological role provides the rationale for B-cell depletion in selected immune-mediated glomerular diseases [1,2,3].

Rituximab, a chimeric type I anti-CD20 monoclonal antibody, remains the most widely used B-cell-depleting therapy in nephrology [4,5]. It acts through complement-dependent cytotoxicity, antibody-dependent cellular cytotoxicity, and direct induction of cell death [6]. In primary membranous nephropathy, rituximab is now one of the main immunosuppressive options for patients at risk of disease progression [4,7,8,9]. It is also used in selected patients with frequently relapsing or steroid-dependent minimal change disease and, less consistently, in focal segmental glomerulosclerosis [10]. Evidence supporting its use in IgA nephropathy remains limited [11,12,13]. In membranoproliferative patterns of injury, treatment is guided by the underlying pathogenic mechanism. Evidence supporting anti-CD20 therapy remains poor, particularly in primary complement-mediated forms, for which current therapeutic strategies increasingly target complement inhibition. Despite its clinical value, rituximab is not effective or suitable for every patient. Some patients fail to achieve remission, whereas others relapse after an initial response. Treatment may also need to be discontinued because of severe infusion reactions or the development of anti-rituximab antibodies [14]. These situations are often grouped under the broad definition of “rituximab failure”, although they do not share the same underlying mechanisms. Several factors may contribute to an inadequate response. In patients with nephrotic syndrome, urinary loss of rituximab may reduce systemic drug exposure [15]. Other proposed mechanisms include incomplete or short-lived B-cell depletion, CD20 internalization or loss, trogocytosis, variability in Fcγ receptor-mediated effector functions, and advanced irreversible glomerular injury [1,16]. Rituximab may also cause clinically significant infusion-related reactions, including fever, chills, rash, dyspnea, and hypotension, which can prevent further administration [14].

The clinical implications vary depending on the underlying mechanisms. Failure to respond to rituximab does not necessarily imply that B-cell depletion is no longer a useful therapeutic strategy [17]. In some patients, the problem may instead be related to the pharmacokinetic, immunogenic, or structural properties of rituximab itself. Another anti-CD20 antibody with distinct biological characteristics may therefore remain effective when rituximab has proven inadequate or can no longer be administered.

Newer anti-CD20 monoclonal antibodies, including ofatumumab, obinutuzumab, ocrelizumab, and ublituximab, differ from rituximab in their degree of humanization, epitope recognition, molecular structure, Fc engineering, and relative recruitment of complement-dependent and cellular effector mechanisms [1]. These differences may influence the depth and duration of B-cell depletion, immunogenicity, and tolerability. They provide a biological rationale for switching to another anti-CD20 agent in patients with an inadequate response to rituximab, treatment-limiting intolerance, or documented anti-rituximab antibodies.

Evidence on this sequential approach remains limited but has grown in recent years. Early reports mainly described individual patients who received another anti-CD20 antibody after rituximab resistance or intolerance. More recently, case series and observational cohorts have reported similar sequential treatment approaches. Most published experience concerns obinutuzumab in primary membranous nephropathy, whereas evidence for other anti-CD20 agents and other primary glomerular diseases remains limited. Although clinical and immunological responses have been reported after switching, the number of treated patients is still small, and the evidence is largely uncontrolled. These observations therefore raise, but do not prove, the possibility that inadequate efficacy may be agent-specific rather than target-specific in selected patients.

Recent systematic reviews have begun to consolidate the evidence for obinutuzumab in membranous nephropathy. Lurie et al. compared obinutuzumab and rituximab in parallel-group cohorts and excluded studies without a control group and those in which obinutuzumab was administered after rituximab [18]. Their analysis therefore addressed the relative efficacy of the two antibodies in independent treatment groups rather than the outcome of sequential treatment in the same patient. Atay et al. used pooled case-level extraction to examine outcomes among patients with membranous nephropathy treated with obinutuzumab [19]. Although this approach provided a more detailed description of individual patients and their previous treatments, it remained limited to obinutuzumab and membranous nephropathy and was not specifically structured around within-patient comparisons by reason for switching from rituximab.

An important clinical question, therefore, remains incompletely answered: what evidence supports the efficacy and safety of switching to another anti-CD20 antibody in a patient who has not responded adequately to rituximab or can no longer receive it because of intolerance or immunogenicity?

We conducted a systematic review of the efficacy and reported safety of non-rituximab anti-CD20 monoclonal antibodies in adults with primary glomerular diseases who had previously received rituximab. The primary synthesis focused on within-patient sequential evidence, defined as the administration of another anti-CD20 antibody after rituximab in the same patient. Outcomes were examined according to the reason for switching, including inadequate efficacy, treatment-limiting intolerance, and documented anti-rituximab antibodies. We considered all eligible non-rituximab anti-CD20 agents and all primary glomerular diseases represented in the available literature. By examining different agents and diseases according to the reason for switching from rituximab, this review addresses a clinical question that previous single-agent, single-disease reviews have not fully answered.

## 2. Materials and Methods

This systematic review was conducted and reported according to the Preferred Reporting Items for Systematic Reviews and Meta-Analyses 2020 statement [20]; the completed checklist is provided in Appendix A. The protocol was prospectively registered in the Open Science Framework (OSF; website https://osf.io/psdk6 accessed on 6 June 2026).

Studies were included if they met the following PICO (population, intervention, comparison, outcome) criteria.

The following eligibility criteria were applied:

- Participants: Adults aged ≥18 years with a primary glomerular disease were eligible, namely membranous nephropathy (MN), minimal change disease (MCD), primary focal segmental glomerulosclerosis (FSGS), IgA nephropathy (IgAN), membranoproliferative glomerulonephritis (MPGN), and C3 glomerulopathy. Mixed-age populations were eligible only when adult data could be extracted separately. Two categories of patients were excluded a priori: secondary forms of glomerular disease and kidney transplant recipients, including patients with recurrent or de novo glomerular disease in the allograft, because the concomitant maintenance immunosuppression and the allograft-specific determinants of proteinuria and graft function would not allow the within-patient comparison of interest to be attributed to the anti-CD20 antibody.
- Intervention: Eligible interventions were anti-CD20 monoclonal antibodies other than rituximab, namely obinutuzumab, ofatumumab, ocrelizumab, ublituximab, veltuzumab, and zuberitamab.
- Comparator. The comparator was previous rituximab treatment in the same patient. Only reports providing a within-patient comparison between the response to rituximab and the response to a subsequently administered non-rituximab anti-CD20 antibody were therefore included. Specifically, comparison between rituximab and new generation of anti-CD20 Ab in different patient groups were not eligible.
- Outcomes: Each report had to provide at least one clinical, biochemical, immunological, or safety outcome measured after the non-rituximab anti-CD20 antibody. Primary efficacy outcomes were complete remission (CR), partial remission (PR), relapse, and treatment resistance. Secondary outcomes were changes in proteinuria, serum albumin, serum creatinine or estimated glomerular filtration rate (eGFR), peripheral B-cell count. In MN, anti-PLA2R antibody titers were also collected when available. Safety outcomes comprised infusion-related reactions, infections and severe infections, hypogammaglobulinemia, neutropenia, hospitalization, treatment discontinuation, serious adverse events, and death. CR and PR were defined according to the definition suggested by KDIGO guidelines [21,22], see further paragraph of outcomes.

Study Designs: Case report, case series, observational studies (prospective cohort studies, retrospective studies) and randomized controlled trials (RCTs), which provided that within-patient data comparing previous treatment with subsequent non-rituximab anti-CD20 therapy were retrievable.

No language restrictions were imposed, and translations were attempted for non-English published articles. No time restriction was applied.

Notably, reports were excluded for any of the following reasons: previous rituximab exposure could not be confirmed; adult data could not be separated from pediatric data; the glomerular disease was secondary; the patient was a kidney transplant recipient; or clinical outcome data following the non-rituximab anti-CD20 treatment were insufficient to classify the response.

### 2.1. Identification and Management of Overlapping Reports

To avoid double-counting, potentially overlapping study populations were identified by comparing study settings, recruitment periods, participating centers, authorship, sample sizes, and participant characteristics. When multiple reports described the same or overlapping case, the most informative report was selected based on completeness of outcome reporting, sample size, and follow-up duration. When overlap was plausible but could not be confirmed, both reports were retained and the uncertainty was flagged in the notes of the Supplementary Materials.

### 2.2. Electronic Searches

The search strategy was developed by FKM and edited by LFS and FN. A search was conducted in PubMed and Scopus (inception to 15 May 2026, with no language restrictions) to identify eligible reports. Reference lists of relevant studies were screened.

Search terms included in Scopus: (TITLE-ABS-KEY (obinutuzumab OR ofatumumab OR ocrelizumab OR ublituximab OR veltuzumab OR zuberitamab) AND (TITLE-ABS-KEY (“membranous nephropathy” OR “membranous glomerulonephritis” OR “minimal change disease” OR “minimal change nephropathy” OR “focal segmental glomerulosclerosis” OR “FSGS” OR “IgA nephropathy” OR “Berger disease” OR “membranoproliferative glomerulonephritis” OR “MPGN” OR “C3 glomerulopathy” OR “primary glomerulonephritis” OR “glomerular disease” OR “glomerulonephritis”)).

### 2.3. Study Selection

Records from both databases were imported into reference manager (EndNote 25, Clarivate, London, UK) and automatically deduplicated. Three independent reviewers (L.F.S, L.C. and A.B.) screened titles and abstracts and independently inspected the full texts of all potentially eligible reports to determine eligibility.

### 2.4. Data Collection

Two reviewers (F.K.M. and L.F.S.) independently extracted data. Disagreements were resolved by discussion and, when necessary, by consultation with a third reviewer (F.N.). Extracted variables covered study identification (first author, year, country, design), disease characteristics (glomerular diagnosis, biopsy findings, baseline eGFR, proteinuria, serum albumin, peripheral B-cell count, anti-rituximab titers, and anti-PLA2R titers (where applicable), previous and concomitant immunosuppressive and supportive treatment, the rituximab regimen and the response to it, the duration of the rituximab response, clinical status before switching, the reason for switching and the possible mechanism of rituximab failure, the non-rituximab anti-CD20 agent and its regimen, follow-up, adverse events, efficacy outcomes and their duration. Antibody assays and their positivity thresholds were recorded as reported in each source publication.

The unit of extraction depended on how the source reported its data: we extracted individual patient data for case reports and case series reporting patients separately, and aggregate data when individual values were unavailable; for cohort and comparative studies, we supplemented aggregate data with any within-patient results reported. For each case or subgroup, we recorded clinical response and its duration after the rituximab course and after the subsequent non-rituximab anti-CD20 treatment. We compared outcomes after the non-rituximab anti-CD20 antibody descriptively with the patient’s previously reported response to rituximab, providing a within-person sequential comparison rather than a controlled crossover comparison. To separate the concise synthesis in the main text from the full extraction dataset, the main outcome tables were limited to the number of eligible patients achieving complete remission, partial remission, no clinical remission, and relapse and, for membranous nephropathy, immunological remission. For safety results only, we reported information from the full cohort when subgroup data were unavailable, but this was clearly identified in the table. We retained detailed baseline and end-of-follow-up values for kidney function, proteinuria, serum albumin, peripheral B-cell counts, anti-PLA2R antibodies, anti-rituximab antibodies, follow-up duration, and time to response in the Supplementary Materials. We did not attribute outcome data from the full study cohort to the rituximab-pretreated subgroup unless subgroup-specific results could be explicitly separated. When a publication reported only a combined CR or PR outcome, the result was retained as combined clinical remission and was not assigned to either CR or PR. Missing or non-evaluable observations were reported as NR and were not included in outcome denominators.

### 2.5. Outcome Definitions

Outcomes were defined or reclassified using KDIGO 2021 [21] and KDIGO 2025 thresholds [22]. The full set of definitions is given in Table 1. Where an original publication applied a different definition, we reclassified the outcome according to the prespecified review definitions; where reclassification was impossible, the study was analyzed according to the original definition, and this was noted in the evidence tables.

For MN, immunological remission was treated as a binary outcome and was defined as anti-PLA2R antibody negativity according to the assay-specific threshold used in each source publication. It was summarized as the number achieving immunological remission over the number of evaluable patients who had positive anti-PLA2R antibodies before the non-rituximab anti-CD20 treatment and an available follow-up measurement. For MPGN and C3 glomerulopathy, no single categorical definition could be applied consistently across the different subtypes; remission, relapse, and resistance were therefore recorded as defined in each original report, and changes in proteinuria, serum albumin, serum creatinine, and eGFR were extracted in parallel to allow a descriptive comparison.

### 2.6. Reasons for Switching from Rituximab

The reason for switching was assigned to one of the following prespecified categories:

1. Rituximab primary non-response defined by absence of PR after an adequately delivered rituximab course and an adequate response-assessment interval.
2. Rituximab partial or incomplete response defined by attainment of PR without CR after an adequately delivered rituximab course and an adequate response-assessment interval.
3. Relapse after rituximab response defined by recurrence of active disease after a documented response to rituximab.
4. Rituximab immunization—Detection of anti-rituximab antibodies, with or without impaired B-cell depletion, accelerated drug clearance, infusion reaction, or loss of clinical efficacy.
5. Rituximab intolerance—A serious or clinically relevant adverse reaction precluding further rituximab administration.
6. Mixed reasons two or more categories in the same group without any detail about their distribution.
7. Other or unclear—A reason outside the preceding categories, or one described too incompletely to be classified.

We extracted and categorized potential biological correlates of rituximab failure, when reported, as failure to achieve peripheral B-cell depletion, early CD19+ B-cell reconstitution, persistent active disease despite sustained or profound peripheral B-cell depletion, or anti-rituximab antibodies. We interpreted these findings as possible mechanisms rather than proven causal explanations. When data were insufficient to assign a mechanism, we classified the case as unclassifiable for mechanism.

### 2.7. Assessment of Heterogeneity

Heterogeneity was assessed by comparing, across studies, the variables listed in the Data Collection section, with particular attention to the underlying glomerular disease, the non-rituximab anti-CD20 agent and its regimen, the reason for switching, the outcome definitions applied, and the duration of follow-up. Given the substantial heterogeneity across the included studies, quantitative synthesis was not performed; findings were synthesized narratively with a table.

### 2.8. Quality and Relevance Assessment

Each report was appraised with the design-specific JBI critical appraisal tool [23]: the checklist for case reports, for case series, for cohort studies, or the relevant quasi-experimental or RCT checklist, as appropriate to the design. Two reviewers, drawn from A.B., L.C., and L.F.S., appraised each study independently, classifying each item as “Yes,” “No,” “Unclear,” or “Not applicable” according to JBI guidance.

No unvalidated numerical summary score was computed, since composite scores conflate items of unequal importance and are not recommended by JBI. Individual checklist judgements were instead reported descriptively and summarized in a dedicated table.

### 2.9. Certainty Assessment

The certainty of evidence was assessed using the GRADE approach for each clinically relevant outcome and separately for each glomerular disease [24]. GRADE judgments were made at the level of the overall body of evidence rather than for individual studies. Evidence derived from observational studies, case series, and case reports started at low certainty and was assessed for risk of bias, inconsistency, indirectness, imprecision, and publication bias. The judgments were based on the direction and consistency of the reported effects, the number of contributing studies and patients, the completeness and duration of follow-up, the comparability of outcome definitions, and the availability of within-patient data after rituximab and subsequent non-rituximab anti-CD20 treatment. Inconsistency was not considered a reason for downgrading when only one study contributed to an outcome or when the available evidence was insufficient to assess between-study variability. Potential overlap between GRADE domains was considered to avoid double downgrading for the same limitation. No upgrading was applied because the uncontrolled study designs, concomitant therapies, selected populations, and potential publication bias precluded a confident attribution of the observed effects to the non-rituximab anti-CD20 agent. Certainty was classified as high, moderate, low, or very low.

### 2.10. Data Synthesis

Studies were grouped by glomerular disease, non-rituximab anti-CD20 agent, reason for switching from rituximab, study design, and level of outcome reporting. Table 2 summarizes the study design, glomerular disease subtype, overall series size, and number of eligible cases, whereas Table 3 summarizes the non-rituximab anti-CD20 regimen, prior and concomitant immunosuppression, rituximab exposure, and the reason for switching. Disease-specific main-text tables provide a concise synthesis of clinical outcomes for membranous nephropathy (Table 4), minimal change disease (Table 5), and focal segmental glomerulosclerosis (Table 6). Detailed clinical and laboratory trajectories are provided in Supplementary Tables S1–S4. Safety outcomes are summarized in Table 7, and GRADE certainty judgments in Table 8.

JBI appraisals and GRADE certainty judgements are presented in separate summary tables.

## 3. Results

**Figure 1:**
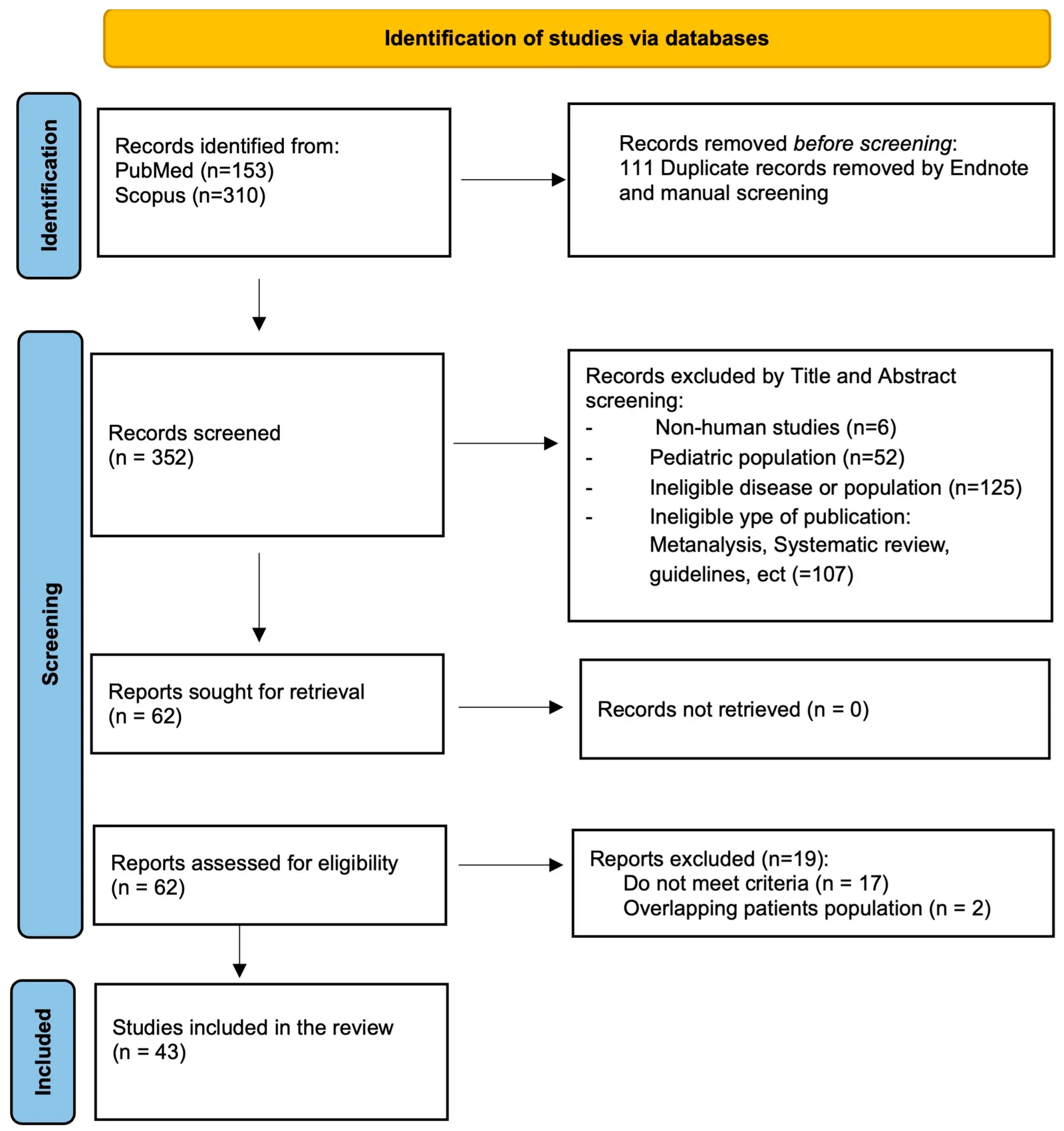
Screening procedure of manuscripts.

Initially, we excluded duplicates as determined using EndNote 2025 and manual screening. In total, 352 manuscripts were identified and screened; 107 were excluded due to the type of publication, 183 were excluded for ineligible population or kidney disease. A total of 62 full-text articles were identified in accordance with the screening criteria, of which 43 were relevant to our focus. Notably, two studies that referred to the same sample were counted only once. The specific procedure for the literature screening is presented in Figure 1.

### 3.1. Study Description

Forty-three studies met the eligibility criteria, comprising 14 retrospective cohort studies, 16 case series, 12 case reports, and one prospective, single-center, single-arm, open-label pilot interventional study. Across these reports, 309 adult patients or individually eligible cases were included in the within-patient synthesis.

We classified patients according to their biopsy-defined glomerular diagnosis. Accordingly, we retained the patient with coexisting IgAN and an MCD-like podocytopathy phenotype within the IgAN group rather than reclassifying them as MCD, to preserve this distinct clinicopathological presentation. We nevertheless considered the MCD-like phenotype separately when interpreting treatment response. Table 2 reports the main characteristics of all included studies.

### 3.2. Other Immunosuppressive Therapy and Reason for Switching to Non-Rituximab Anti-CD20 Antibody

At the patient level, the principal reason for replacing rituximab with a non-rituximab anti-CD20 antibody was classified as primary non-response in 183/309 (59.2%), partial or incomplete response in 4/309 (1.3%), relapse after previous response 29/309 (9.4%) anti-rituximab immunization in 34/309 (11%), mixed or heterogeneous indications in 11/309 (3.6%), rituximab intolerance in 11/309 (3.6%), and unclear or other reason in 37/309 (12%). Table 3 reports previous and concomitant immunosuppression, rituximab response and duration, the clinical phenotype of failure, available biological correlates, and the reason for switching.

### 3.3. Efficacy Results

#### 3.3.1. Membranous Nephropathy

MN represented the largest body of evidence, including seven case reports [31,35,37,48,59,62,64], 11 case series [27,38,40,44,47,50,52,63,66,68,69] 14 retrospective observational cohort studies [11,26,28,29,33,41,43,49,51,53,54,56,57,60], and one prospective single-center, single-arm interventional pilot study [58] for a total of 283 patients. Thirty of 33 reports (90.9%) documented at least one patient achieving CR or PR after switching from rituximab to a non-rituximab anti-CD20 monoclonal antibody.

A sufficiently reliable link between the clinical phenotype that led to stopping rituximab and the outcome after treatment with a non-rituximab anti-CD20 agent can be established based on 148 patient observations from 22 MN reports. Of the 103 patients with a primary non-response to rituximab, 21 had a CR and 53 had a PR. Four more patients achieved a CR or PR but were not reported separately, and 25 did not reach formal clinical remission. One subsequent relapse was recorded. The only patient with a clearly identifiable partial or incomplete response to rituximab achieved a PR after switching treatment. All three patients treated after relapse following an earlier response to rituximab attained a CR. Of the 33 patients switched due to immunization to rituximab, 2 achieved a CR and 29 had a PR, while 2 did not achieve remission. Six further relapses were documented. Of the eight patients switched due to intolerance to rituximab, seven achieved a CR or PR, though the two remission categories could not be distinguished, and one did not achieve formal remission. In other MN studies, either the previous rituximab phenotype was not adequately described, or the post-switch outcomes could not be reliably assigned and were therefore excluded from this phenotype-specific account. Most patients received obinutuzumab, whereas smaller numbers received ofatumumab or zuberitamab. Table 4 summarizes the available clinical and immunological outcomes according to the level of reporting. Detailed patient-level or eligible-subgroup data are presented in Supplementary Table S1, whereas aggregate cohort data are presented in Supplementary Table S2.

#### 3.3.2. Minimal Change Disease

In minimal change disease (MCD) we identified only 18 patients from eight reports [25,27,34,45,46,61,63,70], predominantly case reports and small case series. The median follow-up was 12 months, with reported follow-up ranging from 6 to 36 months. In total, 17 of 18 patients with active disease attained remission: CR was reported in 14/17 patients (83.3%) and partial remission in 3/17 (16.7%), while one maintained the previous CR. Among the nine patients for whom this information was available, the median time to response was 1 month (range, 1–6 months). For eight patients, a reliable connection could be made between the clinical phenotype that led to switching from rituximab and the outcome after treatment with the non-rituximab anti-CD20 agent. The patient with primary non-response to rituximab attained a PR [25]. The one with a partial or incomplete response to rituximab achieved a CR [39]. Of the four patients treated after relapse [27,61] following a previous response to rituximab, all achieved a CR. One of them later relapsed during follow-up [27], corresponding to 5.6% of the overall MCD population and 6.7% of complete responders. The patient who switched due to intolerance to rituximab also achieved a CR [46]. In one case, the patient switched because of rituximab immunization while already in CR and remained in CR after receiving obinutuzumab. This case illustrates maintenance of a pre-existing remission rather than induction of a new remission [34]. In the other ten patients, as reported in the Lin Yuxin [45] and Zamora [63] series, outcomes after switching were favourable, but the individual rituximab phenotypes before the switch could not be reliably linked to disease-specific outcomes. Thus, although all patients with active disease and a definable rituximab phenotype responded after switching, the small number of patients in each phenotype-specific subgroup and incomplete patient-level linkage in the larger series prevent comparison of response rates between phenotypes. Table 5 summarizes the clinical outcomes in MCD. Detailed patient-level baseline and follow-up data, including kidney function, proteinuria, serum albumin, B-cell counts, time to response, and relapse, are provided in Supplementary Table S3.

#### 3.3.3. Focal Segmental Glomerulosclerosis

Evidence in FSGS was restricted to six patients reported across four case reports [30,36] or case series [45,63], with a median follow-up of 15 months (range, 11–26 months). Five of six patients achieved at least an initial clinical response, corresponding to an overall response rate of 83.3%. CR was reported in 4/6 patients (66.7%), while 1/6 (16.7%) achieved partial remission; and no clinical remission in 1/6 (16.6%). Three of the five responders subsequently relapsed [45,63]. In FSGS, the phenotype-based interpretation was more restricted. We could reliably connect the rituximab phenotype that led to treatment switching with the outcome in only two of the six patients. The patient with a primary non-response to rituximab did not achieve a CR or PR after receiving obinutuzumab, despite reduced proteinuria. The patient with a partial or incomplete response to rituximab achieved a CR within about two months and remained in remission during follow-up. Table 6 summarizes the clinical outcomes in FSGS. Detailed patient-level baseline and follow-up data are provided in Supplementary Table S4.

#### 3.3.4. Immunoglobulin A Nephropathy

Only two reports [32,67] illustrated the use of a non-rituximab anti-CD20 antibody in IgAN with 12 months of follow-up. One patient [67] had an MCD-like nephrotic phenotype and achieved CR within approximately 1 month, with consistent drop in proteinuria from 3.8 to 0.3 g/day, serum albumin improvement from 23.7 to 45 g/L, and serum creatinine decreasing from 1.38 to 0.81 mg/dL at first posttreatment assessment. The second case [32] had a proliferative, crescentic IgAN phenotype with partial foot-process effacement and achieved PR approximately 6 months after obinutuzumab with a significant decrease in proteinuria from 4.19 to 0.94 g/day, serum albumin improvement from 33.5 to 37.1 g/L, serum creatinine remained almost stable (from 109 to 97 μmol/L). No relapse was reported during follow-up. Therefore, MDC phenotype should be interpreted as evidence relating to an IgAN-associated podocytopathy and should not be extrapolated to conventional IgAN, while the second one showed a proliferative, crescentic IgAN on a biopsy performed 9 years before anti-CD20 treatment, and should therefore be regarded as anecdotal according to current KDIGO Guidelines [21,22].

#### 3.3.5. Membranoproliferative Glomerulonephritis or C3 Glomerulopathy

No reports were found.

### 3.4. Safety

Safety during administration was reported in 38 of the 43 included reports, whereas five reports provided no information on infusion-related events. In total, 20 reports described infusion reactions, while 18 reports explicitly stated that none occurred. Most reactions were mild or moderate and included chills, fever, rash, pruritus, sweating, nausea, tachycardia or palpitations, throat tightness, rhinitis, dyspnea, wheezing, and bronchospasm. Hypotension and oedema were reported less frequently. Overall, infusion reactions were generally transient and manageable, with no consistent evidence of treatment discontinuation or severe acute toxicity across the available reports.

During follow-up, potentially treatment-related adverse events were reported in 19 studies, two additional studies described events considered unlikely to be treatment-related, 15 reported no events, and seven provided inadequate information. Infections were the most frequently described complications and included upper respiratory and urinary tract infections, pneumonia, COVID-19, bronchitis, herpes zoster or genital herpes, soft-tissue infection, and tuberculosis-test conversion. Hematological or immunological abnormalities included leukopenia, neutropenia, lymphopenia, and hypogammaglobulinemia. Other reported events comprised transient liver dysfunction, gastrointestinal symptoms, musculoskeletal pain, asthenia, and elevated D-dimer levels. Two events—recurrent urinary tract infections in a patient receiving dapagliflozin and coronary artery disease requiring angioplasty—were considered unlikely to be related to anti-CD20 therapy. One cohort also reported deaths during follow-up, although their relationship to treatment was not clearly established. Notably, when subgroup-specific safety data were unavailable, full-cohort safety data were kept as background information and are indicated in Table 7.

### 3.5. Quality Assessment

#### 3.5.1. Case Reports

Twelve case reports were appraised with the JBI checklist for case reports. Overall reporting quality was moderate to high: most reports adequately described patient demographics, clinical history, and current condition, and presented objective, longitudinal outcomes following the non-rituximab anti-CD20 antibody, with several providing biopsy confirmation and extended follow-up [36,37,46,67]. The most consistent methodological weakness was incomplete or unsystematic adverse-event reporting (Q7), which was rated unclear in several reports [30,61,62,67]. Causal attribution was further limited across the group by concomitant immunosuppression—most often corticosteroids or calcineurin inhibitors administered alongside or shortly before obinutuzumab—by pre-emptive or early retreatment, and by the absence of anti-rituximab antibody testing, all of which make it difficult to isolate the effect of the anti-CD20 switch. A minority showed more substantive limitations: Zhang 2024 [64] had diagnostic ambiguity, selective outcome emphasis, and internal inconsistencies between narrative and figures; and Xu 2024 [62] emphasized efficacy despite the absence of a conventional complete or partial remission. The intrinsic single-patient design limited evidentiary weight.

#### 3.5.2. Case Series

Sixteen case series were appraised with the JBI checklist for case series. Reporting of outcomes was generally objective and, in the better series, standardized and longitudinal [40,52,53], but the domains governing internal validity were weak across almost the entire group. Consecutive and complete inclusion (Q4–Q5) were frequently unclear, indicating uncertain patient selection and probable selection bias. Prior therapies and concomitant immunosuppression were heterogeneous and often incompletely reported, and follow-up duration was variable and sometimes short. Several series carried additional, specific threats to validity: Sethi et al. [50] pooled native-kidney and transplant disease with incomplete treatment detail and only partial rituximab exposure; Teisseyre et al. [55] used non-consecutive, biomarker-guided allocation and likely contained two duplicate cases; Chen et al. [71] included two minors; Ding 2025 [32] included only one rituximab-exposed patient; and Zheng et al. [66] had substantial missing outcome and treatment data with internal inconsistencies in age and serologic time points. Safety ascertainment was generally limited. These reports had high susceptibility to selection and reporting bias.

#### 3.5.3. Observational Studies

Fourteen observational studies were appraised with the JBI checklist for cohort studies. These were the methodologically strongest reports in the review, and the best of them combined standardized outcome measurement, complete screening for secondary causes, and adjusted or multivariable analyses [28,49,53]. Nonetheless, all shared the limitations inherent to retrospective, predominantly single-center designs. Recurrent weaknesses were baseline imbalance between groups, incomplete confounding control, and exclusion of patients lost to follow-up, which together bias effect estimates. Identification and handling of confounders (Q4) and completeness of follow-up (Q9) were the domains most often rated unclear or no. The within-patient comparison was weakened in several cohorts by short follow-up after the switch and, in studies enrolling mixed populations, by a rituximab-exposed subgroup whose individual data could not be separated from the overall cohort [28,43,44]. In the multicenter studies, treatment allocation depended on the treating nephrology center and drugs were pooled across agents, introducing center effects and unadjusted confounding that substantially weaken the comparative conclusions [56]. Boyer-Suavet et al. [26] additionally classified exposure after baseline and provided only highly uncertain ofatumumab evidence. Overall, the observational studies were methodologically stronger than the case-level reports, although residual confounding and selection bias remained substantial.

#### 3.5.4. Prospective (Quasi-Experimental) Study

A single prospective study was appraised with the JBI checklist for quasi-experimental studies [58]. Its prospective design, consecutive enrolment, complete follow-up, and objective, repeatedly measured outcomes place it well above the rest of the evidence base in methodological rigor. The principal limitations are structural rather than related to conduct: the uncontrolled single-arm design provides no concurrent comparator (Q4), and the small sample and reliance on historical assumptions about the expected remission rate preclude causal or comparative conclusions. This was the methodologically strongest study included in the review, although its uncontrolled single-arm design precluded causal or comparative conclusions.

All results of JBI checklist were reported in Appendix B.

### 3.6. Certainty

The certainty of evidence was rated as very low for all outcomes. However, the evidence base was substantially more extensive and methodologically developed for membranous nephropathy than for the other glomerular diseases. In MN, clinical and immunological remission were supported by multiple retrospective cohorts and one prospective single-arm study, in addition to case series and case reports. Nevertheless, the absence of a concurrent comparator, residual confounding, heterogeneous follow-up, and potential publication bias prevented a higher certainty rating. Evidence for MCD, FSGS, and IgAN was further limited by very small sample sizes and reliance predominantly on case-level reports. Imprecision was particularly serious for MCD, FSGS, and IgAN because the evidence was based on very small numbers of patients. Publication bias was considered likely because successful or otherwise clinically unusual cases may have been preferentially reported. Evidence concerning relapse and safety was also of very low certainty because follow-up was inconsistent and adverse-event surveillance was frequently incomplete or not systematically reported. Table 8 summarized the GRADE certainty according to the type of nephropathy and to the results.

## 4. Discussion

The present systematic review examined the sequential use of non-rituximab anti-CD20 monoclonal antibodies in 309 adults with primary glomerular diseases previously treated with rituximab. The evidence was heavily concentrated in MN, which accounted for 283 patients, and most patients received obinutuzumab. At the report level, 30 of the 33 studies in MN documented at least one CR or PR. The results outside MN were based on much smaller numbers. Among the 17 patients with active MCD, all achieved a clinical response; the remaining patient received obinutuzumab while already in complete remission. Five of six patients with FSGS achieved an initial response, including four complete and one partial remission; three of the five responders subsequently relapsed. One reported patient with IgA nephropathy had contextually MCD with sign of podocytopathy at biopsy and improved after obinutuzumab with CR outcome. While the other had proliferative and crescentic disease at biopsy with partial foot-process effacement and achieved only PR. These observations therefore cannot be considered evidence of efficacy in conventional IgAN, according to KDIGO guidelines [21,22]. The safety findings were broadly consistent with the known effects of B-cell depletion. Infusion reactions were usually mild or moderate and manageable, while infections were the most frequently reported events during follow-up. These apparently favorable results need to be balanced by the quality of evidence. About the quality of the evidence, we highlight certainty remained very low according to GRADE in all nephropathies, but the evidence base was not equally limited across diseases: MN was supported by a substantially larger number of patients, several retrospective cohorts, and one prospective single-arm study, whereas evidence for MCD, FSGS, and IgAN were supported by small case series and individual reports. Thus, the GRADE category should not be interpreted as indicating equivalent evidentiary support across all glomerular diseases. Rather, MN represents the most developed, although still uncontrolled and potentially biased, body of evidence.

The present review differs from the available evidence syntheses in a fairly important way. Lurie et al. [18] compared obinutuzumab and rituximab in parallel treatment groups and excluded studies in which obinutuzumab was administered after rituximab. Their meta-analysis therefore addressed whether the two drugs produced different outcomes in different patients, rather than whether changing the antibody could rescue the same patient after rituximab failure [19]. Atay et al. used pooled case-level data, which allowed a more detailed description of individual responses, but their analysis remained restricted to obinutuzumab in membranous nephropathy. Earlier reviews of second- and third-generation anti-CD20 antibodies had an even broader immunological scope and were not built around the clinical problem of treatment sequencing in glomerular disease. Our eligibility framework covered the major primary glomerular diseases and all available non-rituximab anti-CD20 antibodies. In practice, eligible evidence was found only for membranous nephropathy, minimal change disease, focal segmental glomerulosclerosis, and IgA nephropathy. More importantly, the previous rituximab course was the comparator for each patient. This within-patient approach does not replace a randomized comparison, but it answers a different question: what clinical and safety outcomes have been reported after sequential switching from rituximab to another anti-CD20 antibody?

An important feature of this review was the explicit separation of clinically distinct reasons for switching from rituximab. We specifically retained primary non-response, partial/incomplete response, relapse after a previous response, immunization, and intolerance as different phenotypes. This distinction is clinically relevant because the meaning of a post-switch CR or PR depends on the pre-switch state. Thus, our analysis may provide a clinical framework for interpreting the rationale for switching from Rituximab. Unfortunately, the available data seem fragmentary regarding the underlying mechanisms and provide little support for guiding clinical decisions according to the different rituximab-response phenotypes. However, patients who stopped rituximab because of intolerance showed particularly consistent responses after another anti-CD20 antibody. In Podesta et al. [49], all seven rituximab-intolerant patients achieved CR or PR after ofatumumab, compared with three of ten patients with primary non-response. A complete remission was also reported by Loeffler et al. [46] after life-threatening laryngospasm during rituximab infusion. Rituximab immunization is a clinical situation in which switching to a different anti-CD20 antibody may be considered. Among 33 patients with MN who switched because of anti-rituximab antibodies, 31 achieved clinical remission after receiving a non-rituximab anti-CD20 agent, although some patients later relapsed. These findings suggest that rituximab immunization may represent a clinically relevant reason for treatment change. However, the presence of anti-rituximab antibodies does not necessarily imply a neutralizing mechanism or indicate that immunization is the sole cause of rituximab failure. Finally, patients who switched after relapse following a previous rituximab response also appeared to respond favorably to alternative anti-CD20 therapy. All three MN patients with a reliable phenotype–outcome linkage achieved CR after switching. This may reflect preserved sensitivity to CD20-directed therapy, while differences in their pharmacological properties and mechanisms of B-cell depletion could also contribute, although the available data do not allow us to distinguish these possibilities. These observations are based on small numbers and do not provide a reliable comparative estimate; future reports should take this aspect into account.

In cases of primary non-response after a properly administered course of rituximab, the situation was more complicated. Responses after switching were variable but observed in several cohorts, especially among patients with MN who received obinutuzumab. The pharmacological properties of obinutuzumab could contribute to more extensive or qualitatively different B-cell depletion [72,73,74,75]. However, delayed effects of rituximab, retreatment intensity, concomitant immunosuppression, and spontaneous variation in proteinuria could also explain the observations. Furthermore, in many cases, researchers did not measure anti-rituximab antibody levels, serum rituximab concentration, or duration of B-cell depletion, making clinical conclusions difficult.

The safety signal was reassuring in some respects but not strong enough to establish that newer antibodies are safer than rituximab. Among the 38 reports providing infusion-safety information, 20 described infusion reactions, which were generally transient and manageable. Infections were the main events during follow-up. Hypogammaglobulinemia, neutropenia, lymphopenia, herpes virus infections, pneumonia, and urinary tract infections were reported, although not consistently sought or defined. Several publications offered little more than a statement that no adverse events occurred. This is not equivalent to structured safety surveillance. It is particularly relevant in patients receiving repeated anti-CD20 courses, concomitant corticosteroids or calcineurin inhibitors, and in those with reduced kidney function. The available data therefore support feasibility, but they do not allow a reliable comparison of severe infection, persistent hypogammaglobulinemia, or late adverse events between obinutuzumab, ofatumumab, and rituximab.

Important limitations stemmed from both the underlying evidence base and the review process itself. No randomized trial examined switching from rituximab to another anti-CD20 antibody. Most evidence came from retrospective cohorts, case series, and selected case reports, with a considerable risk of publication bias. The literature was dominated by obinutuzumab and MN, while information on other antibodies and glomerular diseases was limited. There were substantial differences in doses used, retreatment strategies, rituximab failure phenotypes, and length of follow-up. Concomitant immunosuppression was frequently used. At the review stage, aggregate reporting often made it impossible to reclassify patients individually. Different phenotypes could not always be distinguished, and some residual overlap between reports cannot be ruled out. The biological features of rituximab failure could not be fully characterized because anti-rituximab antibodies, drug exposure, urinary loss, and the extent and duration of B-cell depletion were seldom assessed together. Due to these limitations, it is not possible to draw causal conclusions about why switching succeeded or failed in individual patients.

Future studies should separate rituximab intolerance, immunization, incomplete B-cell depletion, relapse after response, and true refractory disease from the outset. These categories should not be pooled under a generic definition of rituximab failure. Prospective multicenter studies should use disease-specific KDIGO outcomes, predefined response intervals, standardized anti-PLA2R and B-cell monitoring, and longer safety follow-up. Measurement of anti-rituximab antibodies and, where feasible, rituximab exposure would also help to identify patients in whom changing the antibody is more rational than abandoning CD20-directed therapy altogether.

## 5. Conclusions

Non-rituximab anti-CD20 antibodies may represent an option in selected patients previously treated with rituximab, although the available evidence remains of very low certainty. In rituximab-intolerant patients, early reports suggest that switching to another anti-CD20 antibody can be effective when further rituximab treatment is not feasible, but supporting evidence remains limited. Favorable responses have also been reported in patients with anti-rituximab immunization and in those who relapsed after a previous rituximab response. However, these observations are based on small numbers and low-certainty evidence. Primary non-response remains the most uncertain setting, with variable outcomes after switching and limited information on the mechanisms underlying rituximab failure. Relapses after newer anti-CD20 antibodies were also observed, indicating that an initial response does not necessarily translate into durable disease control; overall, the current data suggest that the reason for switching may be clinically relevant, but are insufficient to define clear phenotype-based treatment recommendations.

## Figures and Tables

**Table 1:** Prespecified disease-specific definitions of treatment response.

Disease	Complete Remission	Partial Remission	Relapse	Resistance
Membranous nephropathy	Proteinuria ≤ 0.3 g/day or uPCR ≤ 300 mg/g, normal serum albumin, stable or preserved kidney function	Proteinuria ≤ 3.5 g/day with ≥50% reduction from baseline, normalized or clearly improved serum albumin, stable kidney function	Reappearance of nephrotic-range proteinuria (>3.5 g/day or equivalent uPCR) after CR or PR	Failure to reach CR or PR after an adequately delivered course and an adequate disease-specific assessment interval
Minimal change disease and primary FSGS	Proteinuria ≤ 0.3 g/day or uPCR ≤ 300 mg/g, normal serum albumin, stable or preserved kidney function	Sub-nephrotic proteinuria reduced ≥50% reduction in proteinuria from baseline and proteinuria ≤ 3.5 g/d (or equivalent), with stable kidney function, serum albumin improvement may be reported as supportive evidence	Reappearance of nephrotic-range proteinuria or of nephrotic syndrome after CR or PR	As above
IgA nephropathy ^a^	Review-defined complete proteinuria response: Proteinuria ≤ 0.5 g/day (ideally ≤ 0.3 g/day) with stable eGFR	Review-defined complete proteinuria response: Proteinuria ≤ 1 g/day (or uPCR ≤ 1000 mg/g) with stable kidney function	Reappearance or clinically relevant sustained increase in proteinuria and/or hematuria after documented remission	As above
MPGN and C3 glomerulopathy	As defined in the original report *	As defined in the original report *	As defined in the original report *	As defined in the original report *

Footnotes: CR, complete remission; eGFR, estimated glomerular filtration rate; FSGS, focal segmental glomerulosclerosis; MPGN, membranoproliferative glomerulonephritis; PR, partial remission; uPCR, urinary protein-to-creatinine ratio. ^a^ For IgA nephropathy, proteinuria response categories were defined for the purposes of this review and adapted from KDIGO 2025 treatment targets; they should not be interpreted as formal KDIGO complete or partial remission categories [22], * Continuous variables (proteinuria, serum albumin, serum creatinine, eGFR) were extracted in addition.

**Table 2:** Principal characteristics of the studies.

Study-ID	Country/Year	Design (Report/Series)	GN Subtype	Series Size (Number of Included Cases)
Angeletti.Andrea.2025. [25]	Italy/2025	Case series	MCD	6 (1)
Boyer-Suavet.Sonia.2020. [26]	United States/2020	Retrospective cohort	MN	44 (3)
Chen.Zewei.2025. [27]	China/2025	Case series	MN, MCD	12 (10)
Cheng.Xi.2025. [28]	China/2025	Retrospective cohort	MN	55 (30)
Cheng.Xueyang.2025. [29]	China/2025	Retrospective cohort	MN	74 (4)
ChiliquingaMorales.Byron.2025. [30]	Spain/2025	Case report	FSGS	1 (1)
deLaPradaAlvarez.FranciscoJose.2024. [31]	Spain/2024	Case report	MN	1 (1)
Ding.Qingyun.2025. [32]	China/2025	Case series	IgAN	3 (1)
Dong.Rui.2025. [33]	China/2025	Retrospective cohort	MN	15 (11)
Gauci.Pierre-Alexis.2025. [34]	France/2025	Case series	MCD	3 (1)
Guo.Jianying.2026. [35]	China/2026	Case report	MN	1 (1)
Henriques.AndreiaRita.2026. [36]	Portugal/2026	Case report	FSGS	1 (1)
Hudson.Rebecca.2022. [37]	Australia/2022	Case report	MN	2 (2)
Jha.VijoyKumar.2025. [38]	India/2025	Case series	MN	6 (6)
Jin.Li.2024. [39]	China/2024	Case series	MCD	6 (1)
Klomjit.Nattawat.2020. [40]	United States/2020	Case series	MN	3 (3)
Li.Huxian.2026. [41]	China/2025	Retrospective cohort	MN	17 (17)
Li.Luyao.2025. [42]	China/2025	Retrospective cohort	MN	25 (21)
Li.Xue-Qi.2025. [43]	China/2025	Retrospective cohort	MN	25 (2)
Lin.Yuxin.2024. [44]	China/2024	Case series	MN	18 (12)
LinYuxin.2025. [45]	China/2025	Case series	MCD, FSGS	11 (11)
Loeffler.Thomas.2025. [46]	France/2025	Case report	MCD	2 (1)
Liu.Yang.2026. [47]	China/2026	Case series	MN	6 (6)
Naik.Sachin.2023. [48]	India/2023	Case report	MN	2 (2)
Podesta.ManuelAlfredo.2024. [49]	Italy/2023	Retrospective cohort	MN	17 (17)
Sethi.Supreet.2020. [50]	United States/2020	Case series	MN	10 (6)
Shi.Guo-Qing.2025. [51]	China/2025	Retrospective cohort	MN	75 (5)
Sridharan.Krita.2025. [52]	Australia/2025	Case series	MN	5 (3)
Su.Wenyan.2025. [53]	China/2025	Retrospective cohort	MN	20 (20)
Su.Xiaole.2024. [54]	China/2024	Retrospective cohort	MN	59 (14)
Teisseyre.Maxime.2021. [55]	France/2021	Case series	MN	8 (4)
Teisseyre.Maxime.2025. [56]	France, India, Italy/2025	Retrospective cohort	MN	34 (15)
Tian.Li.2026. [57]	China/2026	Retrospective cohort	MN	25 (25)
Trillini.Matias.2026. [58]	Italy/2026	Prospective, single-center, single-arm, open-label pilot interventional study	MN	20 (20)
Wang.Jinmeng.2025. [59]	China/2025	Case report	MN	2 (2)
Wang.Linna.2026. [60]	China/2026	Retrospective cohort	MN	60 (9)
Wang.Qiang.2024. [61]	China/2024	Case report	MCD	1 (1)
Xu.Qiuyu.2024. [62]	China/2024	Case report	MN	1 (1)
Zamora Jorge Ivan.2025. [63]	Spain/2025	Case series	MN, MCD, FSGS	6 (4)
Zhang.Yao.2024. [64]	China/2024	Case report	MN	1 (1)
Zhao.Xiangling.2026. [65]	China/2026	Case series	MN	31 (9)
Zheng.Zhenfeng.2023. [66]	China/2023	Case series	MN	3 (3)
Zhu.Shuhua.2026. [67]	China/2026	Case report	MCD with coexisting IgAN	1 (1)

Footnotes: MN, membranous nephropathy; MCD, minimal change disease; FSGS, focal segmental glomerulosclerosis; IgAN, IgA nephropathy.

**Table 3:** Previous and/or concomitant immunosuppressive treatment and reason for switching from rituximab.

Study-ID	Type of GN	Previous Cumulative Dose of RTX	Other Previous Immunosuppression	Clinical Response to RTX	Duration of RTX Response	Mechanism of RTX Failure	Phenotype of RTX Failure	Anti-CD20 Agent Dose and Schedule	Concomitant IS
Angeletti.Andrea.2025. [25]	MCD	One course of RTX, dose NR	None	No PR or CR	None	Persistent active disease despite B-cell depletion	Primary non-response	Obinutuzumab, 375 mg/1.73 m2 IV × 2 doses, administered 2 weeks apart	None
Boyer-Suavet.Sonia.2020. [26]	MN	#1 and #2: 4000 mg; #3: 2000 mg	NR	#1: no PR or CR, #2: no PR or CR, #3: Relapse	#1: none, #2: none, #3: 48 m	Documented neutralizing anti-RTX antibodies causing treatment failure	#1, #2, #3: RTX Immunization	Ofatumumab, 300 mg on day 1 and 1000 mg on day 8, with an optional 1000-mg dose on day 21	none
Chen.Zewei.2025. [27]	MN, MCD	Between 2000 and 3000 mg	MCD: CS; CNI; cyclophosphamide	MN: No CR or PR MCD: 3 relapse	MN: none MCD: >6 m	2 MN + 1 MCD: B-cell reconstitution, anti-RTX NR 1MCD: B-cell reconstitution, anti-RTX- 2 MN: B-cell reconstitution + anti-RTX Ab+ 2 MN: Persistent disease despite B-cell depletion; anti-RTX− 1MN + 1 MCD: Persistent disease despite B-cell depletion; anti-RTX NR	Mixed 7 MN: primary non-response 3 MCD: CNI dependence after RTX	Obinutuzumab 1000 mg IV at months 0, 6, 12 and 18	MCD: 2 CNI discontinued
Cheng.Xi.2025. [28]	MN	2 courses of RTX but doses not reported	Unclear/NR Reported only alternative therapy (40 pts): CNI 23/40 (57.5%), cyclophosphamide 9/40 (22.5%), and other agents (tripterygium wilfordii, leflunomide, or MMF) 10/40 (25.0%)	No clinical response without details	NR	RTX resistance with unsatisfactory clinical response	Likely, primary non-response	Obinutuzumab 1000 mg on day 1 and on day 15	Reported only alternative therapy (40 pts): 17/40 were still receiving their previous immunosuppressive regimen
Cheng.Xueyang.2025. [29]	MN	2000 mg + 375 mg/m^2^	NR	No CR or PR	6 m	RTX immunization (anti-RTX antibodies) with loss of efficacy,	Immunization	Obinutuzumab 1000 mg on days 1 and 15, if no CR other course of 375 mg/m^2^	NR
ChiliquingaMorales.Byron.2025. [30]	FSGS	2000 mg	Previous RTX: CS, cyclophosphamide. During RTX: MMF, CNI	No CR or PR	None	Persistent active disease despite documented B-cell depletion	Primary non-response	Obinutuzumab, 1000 mg on days 1 and 15, plus 1000 mg during F-U	CNI
deLaPradaAlvarez.FranciscoJose.2024. [31]	MN	approximately 5000 mg	MMF, cyclophosphamide	No CR or PR	None	NR	Primary non-response	Obinutuzumab 100 mg IV and 900 mg IV on consecutive days, followed by 1000 mg IV 14 days later	NR
Ding.Qingyun.2025. [32]	IgAN	2000 mg	CS, MMF CNI	No CR or PR	None	with B-cell reconstitution at 6 m	Primary non-response	Obinutuzumab, 1000 mg IV as a single dose	None
Dong.Rui.2025. [33]	MN	Dose NR, RTX in 11/15 (73.3%)	Cyclophosphamide plus CS in 7/15 (46.7%) and a CNI plus CS in 2/15 (13.3%)	NR	NR	NR	Unclear: unsatisfactory response to prior immunosuppression	Obinutuzumab cumulative dose in whole cohort: 1 g in 7/15 (46.7%), 2 g in 6/15 (40%), and 3 g in 2/15 (13.3%)	NR
Gauci.Pierre-Alexis.2025. [34]	MCD	2000 mg, administered 18 months before pregnancy	CS, MMF	CR	OBI used as maintenance	RTX immunization: prior RTX remission, switched to obinutuzumab before pregnancy.	Immunization	Obinutuzumab 1000 mg total before pregnancy	CS
Guo.Jianying.2026. [35]	MN	3000 mg	NR	No CR or PR	None	RTX immunization	Immunization	Obinutuzumab 1000 on days 1 and 15	NR
Henriques.AndreiaRita.2026. [36]	FSGS	2500 mg	CS, CNI, and supportive therapy including dapagliflozin	PR	6 m	Persistent disease despite complete peripheral B-cell depletion; B-cell repopulation at ~6 m	Partial/incomplete RTX response	Obinutuzumab, 1000 mg IV twice, 2 weeks apart (initial cumulative dose 2000 mg); an additional 1000 mg IV was administered 10 months	CS and CNI were gradually tapered until discontinuation
Hudson.Rebecca.2022. [37]	MN	2000 mg	NR	#1: No CR or PR #2: PR	#1: None, #2: 9 m	1 with CD19 reconstitution after first RTX + active disease despite profound depletion after repeat RTX. 1 NR	Mixed: 1 primary non-response and 1 Partial/incomplete response	Obinutuzumab 100 mg on day 1, 900 mg on day 2 and 1000 mg on day 16 or day 21	cyclophosphamide with CS
Jha.VijoyKumar.2025. [38]	MN	At least 2000 mg	#1: CS plus CNI #2–3: CNI #4–5–6: CS plus CNI, 6 months of CS plus cyclophosphamide	5 with No CR or PR 1 relapse after PR	8 m	NR	Mixed: 5 primary non-response 1 relapse	Obinutuzumab 1000 mg IV in two doses approximately 14–15 days apart	None
Jin.Li.2024. [39]	MCD	1600 mg	CS	PR	4 m	Persistent incomplete disease despite B-cell depletion	Partial/incomplete response	Obinutuzumab, 1000 mg IV as a single dose	CS
Klomjit.Nattawat.2020. [40]	MN	#1, #3: 4000 mg #2: 2000 mg	NR	No CR or PR	None	2 with B-cell reconstitution, 1 NR	Primary non-response	Obinutuzumab 1000 mg on day 1 and 900 mg on day 2	None
Li.Huxian.2026. [41]	MN	2000 mg	7/17 (41.1%) had previous cyclophosphamide, and/or CNI but concomitant RTX is not established	No CR or PR	6 m	Inadequate or incomplete clinical response	Primary non-response	Obinutuzumab 1000 mg on day 1 and on day 15 in 16 patients, 1000 mg on day 1 in 1 patients. Additional 1000 mg was administered 6 months in 9 patients	None
Li.Luyao.2025. [42]	MN	21/25 in RTX, NR	Whole-cohort previous therapy: CS plus cyclophosphamide 9/25 (36%); CS plus CNI 10/25 (25%); CNI 15/25 (60%), MMF1/25 (4%); immunoglobulin 1/25 (4%)	NR	NR	NR	Other/unclear: primary NR vs. relapse not separable	Obinutuzumab 1000 mg per course, 3/25 (12%) CD 1000 mg, 12/25 (48%) CD 2000 mg, 8/25 (32%) CD 3000 mg, 2/25 (8%) CD 4000 mg	NR/unclear: description in all cohort: None 12/25 (48%), CS 1/25 (4%), CNI 9/25 (36%), CNI plus CS 3/25 (12%)
Li.Xue-Qi.2025. [43]	MN	2000 mg	None	No formal CR/PR	None	Persistent active disease despite peripheral B-cell depletion	Primary non-response	Obinutuzumab 1000 mg on day1, and 1000 mg on day 15 or 22	None
Lin.Yuxin.2024. [44]	MN	12/18 (66.7%) median course of 2.0 (1.3–4.8)	In whole cohort: CS 14/18 (77.8%), cyclophosphamide 8/18 (44.4%), MMF 6/18 (33.3%) CNI 6/18 (33.3%)	NR in RTX group	NR in RTX group	11/12 unsatisfactory response or insufficient maintenance of efficacy and 1/12 RTX infusion reaction	Mixed (11 not separable in primary non response, partial response, relapse/1 Intolerance)	Obinutuzumab 2.0 g (IQR 1.3–3.0)	In whole cohort: 16/18 (88.9%) none, 1/18 (5.5%) CNI, 1/18 (5.5%) cyclophosphamide and CS
Lin.YuxinY.2025. [45]	MCD, FSGS	2400 (IQR 1800–3600)	5/11 (45.4%) none, 4/11 (36.3%) CS, 2/11 (18.2%) CNI	7/11 CR 3/11 PR 6 relapses	NR, 4/7 with CR were classified as short/insufficient maintenance	NR, lack of information about anti-RTX antibodies or B-cell depletion	Mixed: 10/11 relapse 1/11 incomplete/partial response	Obinutuzumab, 1000 mg per infusion, with individualized number and interval of administrations	3 (27%) only CS, 6 (54.5%) CS plus CNI
Liu.Yang.2026. [47]	MN	Median 2000	None, CS plus cyclophosphamide or CNI	5/6 No CR or PR 1/6 PR	5/6: none 1/6: 9 m	Detection of anti-RTX antibodies	Immunization	Obinutuzumab 1000 mg on day 1, and 1000 mg on day 15	None
Loeffler.Thomas.2025. [46]	MCD	Exact RTX dose was NR., 3 course	repeated high-dose CS; long-term CNI; MMF	CR	36 m	Hypersensitivity, life-threatening laryngospasm during RTX infusion	Intolerance	Obinutuzumab 1000 mg IV on day 1 and day 15; cumulative dose 2000 mg	CS and CNI were gradually tapered until discontinuation
Naik.Sachin.2023. [48]	MN	Two RTX administrations but doses not reported	CNI and/or cyclophosphamide plus CS	No CR or PR	None	NR	Primary non-response	Obinutuzumab 1000 mg IV on day 0 and day 15	None
Podesta.ManuelAlfredo.2024. [49]	MN	Median number of RTX infusion 2 with range 1–7	CS 12/17 (70.6%), alkylating agents 11/17 (64.7%), CNI7/17 (41.2%) and MMF 3/17 (17.6%)	No CR or PR	In primary non-response: none. In intolerant patient: NR	10/17 likely, incomplete peripheral B-cell depletion. RTX intolerance: 7/17 hypersensitivity	Mixed: 10/17 Primary non-response 7/17 Intolerance	Ofatumumab median 300 mg (IQR 100–300) in whole cohort	None
Sethi.Supreet.2020. [50]	MN	4/6 of primary MN (66.6%)	none or CNI, CS, and/or MMF	5/6 Unclear not documented CR/PR 1/6 CR or PR	NR	In whole cohort: 5/6 NR 1/6 hypersensitivity with infusion-related anaphylaxis	Mixed: 5/6 unclear-author defined RTX refractory, and 1/6 Intolerance	Obinutuzumab 1000 mg on day 1 and 1000 mg on day 15	NR
Shi.Guo-Qing.2025. [51]	MN	NR for the 5 patients	NR for the 5 patients	2/5 CR or PR 3/5 No CR or PR	2/5 NR 3/5 None	NR	Mixed: 3/5 primary non-response 2/5 relapse	Obinutuzumab 1000 mg on day 1 and 1000 mg on day 15	NR for the 5 patients
Sridharan.Krita.2025. [52]	MN	2000 mg	#1 CNI, #2 modified Ponticelli regimen, #3 Previous CS plus AZA, CNI plus CS	1/3 No CR or PR 2/3 relapse	1/3 none 1/3 3 y 1/3 1 y	NR	Mixed: 1/3 primary non-response 2/3 relapse	Obinutuzumab 100 mg dose on day 1, 900 mg on day 2, and 1000 mg on day 15	CNI or none
Su.Wenyan.2025. [53]	MN	2000 mg	CS 12/20 (60%), CNI 11/20 (55%), cyclophosphamide 5/20 (25%) and MMF 1/20 (5%).	No CR or PR	None	NR	Primary non-response	Obinutuzumab 1000 mg on day 1 and 1000 mg on day 15, plus 1000–2000 mg if proteinuria was >3.5 g/d at 6 months	None
Su.Xiaole.2024. [54]	MN	14/39 (35.9%) median 4.5 doses (IQR 3.25–6.0).	In the whole cohort: CS 10/14 (71.4%), CNI 7/14 (50.0%), cyclophosphamide 3/14 (21.4%), MMF 1/14 (7.1%), and triptolide 1/14 (7.1%)	No CR or PR	None	NR	Primary non-response	Obinutuzumab 1000 mg on day 1 and 1000 mg on day 15–21. Later doses were guided by B-cell count and clinical judgment	NR
Teisseyre.Maxime.2021. [55]	MN	#1–3–4: 3000 mg #2: 2000 mg	Only #4 CNI	3/4 No CR or PR 1/4 PR	3/4 None 1/4 6 m	Documented neutralizing anti-RTX antibodies	Immunization	2/4 (50%) Obinutuzumab 100 mg day 1, 900 mg day 2, and 1000 mg day 8 2/4 (50%) Ofatumumab 300 mg on day 1 and 1 g on day 8	NR
Teisseyre.Maxime.2025. [56]	MN	median 2 g (IQR 2–4)	NR	10/15 primary non-response 5/15 relapse in CR or PR	10/15 None 5/15 NR	Documented neutralizing anti-RTX antibodies	Immunization	12/15 Obinutuzumab 100 mg IV day 1, 900 mg day 2, and 1000 mg on days 8 and 15 3/15 ofatumumab 300 mg IV day 1 and 1000 mg day 8 (one patient also received 1000 mg on day 21)	None
Tian.Li.2026. [57]	MN	2000 mg (IQR 2000–4000)	CS plus cyclophosphamide in 8/25 (32%); CNI with or without CS in 18/25 (72%); at least two immunosuppressive agents in 20/25 (80%)	20/25 No CR or PR 5/25 PR	20/25 None 5/25 NR	NR	Mixed: 20/25 primary non-response 5/25 relapse	Obinutuzumab 1000 mg on day 1 and 1000 mg on day 15–30, further 1000 mg on day 180 according to clinical and laboratory findings	NR
Trillini.Matias.2026. [58]	MN	NR	CS in 10/20 (50%), alkylating agents in 9/20 (45%), CNI in 4/20 (20%), and apheresis in 2/20 (10%), in different combinations. In total, 5/20 (25%) patients had received ofatumumab and one had received felzartamab infusions; none benefited.	No CR or PR	None	20/20 active disease despite complete peripheral B-cell depletion; 3/20 also had RTX side effects	Mixed: 20/20 non-primary response, 3/20 also intolerant	Obinutuzumab 100 mg on day 1 and 900 mg on day 2, 1000 mg on day 15 and on day 30	None
Wang.Jinmeng.2025. [59]	MN	#1: 6400 mg #2: 6400 mg	#1 CS; CNI; cyclophosphamide; MMF #2: CNI	#1: No CR or PR #2 relapse	#1: None #2: 6 m	Persistent active disease despite peripheral B-cell depletion	Mixed: #1: Primary non-response #2: Relapse	#1: Obinutuzumab 1000 mg on day 1 and 1000 mg on day 8. Then 1000 mg on day 300 #2: Obinutuzumab 1000 mg on day 1 and 1000 mg on day 8	None
Wang.Linna.2026. [60]	MN	2000 or 3000 mg	None, or CS, or CNI or CS plus cyclophos phamide or CS plus MMF	7/9 No CR or PR 2/9 Relapse	7/9 None 2/9 NR	NR	Mixed: 7/9 Primary non-response 2/9 Relapse	Zuberitamab 1000 mg on day 1 and on day 15 or 30. According to clinical condition further infusion (2000–3600 mg)	NR
Wang.Qiang.2024. [61]	MCD	approximately 5000 mg	CS; CS plus CNI	CR	6 m	Relapse associated with B-cell reconstitution	Relapse followed by inadequate response	Obinutuzumab 1000 mg IV as a single infusion	NR
Xu.Qiuyu.2024. [62]	MN	5000 mg	CS and cyclophosphamide	No CR or PR	None	NR	Primary non-response	Obinutuzumab 1000-mg on day 1	CS, and cyclophosphamide
Zamora Jorge Ivan.2025. [63]	MN, MCD, FSGS,	NR	NR	No CR or PR Relapse	3/4: none 1/4: NR	Unclear, 3/4 likely failure to achieve complete peripheral B-cell depletion author suggestion	Mixed: 3/4 primary non-response 1/4 relapse	Obinutuzumab, dose NR	NR
Zhang.Yao.2024. [64]	MN	2500 mg	CS plus CNI; CS plus CNI plus cyclophosphamide, CS plus MMF	No CR or PR	None	Side effect: severe symptomatic hypokalemia	Intolerance	Obinutuzumab 2000 mg over 1st month, 2000 mg over 6th month	None
Zhao.Xiangling.2026. [65]	MN	NR	NR	No CR or PR	None	NR	Primary non-response	Obinutuzumab 1000 mg infusions at weeks 0, 2, and 24	None
Zheng.Zhenfeng.2023. [66]	MN	#1: 4400 mg. #2: 2100 mg. #3: 2000 mg plus an additional unknown dose	#1 case CNI, then CS plus cyclophosphamide	No CR or PR	None	NR	Primary non-response	Obinutuzumab #1: 1000 mg #2: 1000 mg on day 1, on day 90 and on day 180	None, but #2 and #3 had belimumab before switch
Zhu.Shuhua.2026. [67]	MCD with coexisting IgAN	4200 mg	CS, leflunomide, CNI	Relapses after CR	3 y	B-cell reconstitution	Relapse	Obinutuzumab 1000 mg once	

Footnotes: NR, not reported; MN, membranous nephropathy; MCD, minimal change disease; FSGS, focal segmental glomerulosclerosis; IgAN, IgA, nephropathy; RTX, rituximab; CS, Corticosteroids; CNI, Calcineurin inhibitors; MMF, mycophenolate mofetil.

**Table 4:** Clinical outcomes after non-rituximab anti-CD20 in MN.

Study-ID	Patients Assessed (n)	Clinical Status Before Switching	Clinical CR (n)	Clinical PR (n)	No Clinical Remission (n)	Relapse (n)	Baseline PLA2R Positive (n)	Immunol. Remission (n/N Evaluable)	Follow-Up	Time to First CR/PR	Duration of CR/PR
**Reports with patient-level or eligible-subgroup outcome data**											
Boyer-Suavet.Sonia.2020. [26]	3	NR	0	3	0	0	3	3/3	30 m	<3 m	3–30 m
Chen.Zewei.2025. [27]	7	Heavy NS	2	4	1	0	6	5/6	6–24 m	1–10 m	5.5–14 m
Cheng.Xueyang.2025. [29]	4	non-N U-prot	1	3	0	0	NR	NR	6–9 m	3 m	PR 3–6 CR NR
deLaPradaAlvarez.FranciscoJose.2024. [31]	1	NS + KF impairment	0	1	0	0	1	1/1	9 m	6 m	≈3 m
Guo.Jianying.2026. [35]	1	NS	0	1	0	0	1	1/1	≈18 m	≈8 m	≈10 m
Hudson.Rebecca.2022. [37]	2	Heavy NS	0	1	1	0	2	2/2	#1: 15 m #2: 12 m	#1: NA #2: 9 m	#1: NA #2: 3 m
Jha.VijoyKumar.2025. [38]	6	5 with heavy NS, 1 with NS	4	1	1	0	5 (1 NR)	3/3	10–18 m	3–6 m In Resp	CR 10–13 m PR 10 m
Klomjit.Nattawat.2020. [40]	3	Heavy NS	0	2	1	0	3	2/3	9–24	PR: 12 And 18	NR: never PR: 6 m
Li.Xue-Qi.2025. [43]	2	Heavy N U-prot	0	1	1	0	2	0/2	#1: 7 m, #2: NR	#1: 1 m #2: NR	NR
Liu.Yang.2026. [47]	6	4/6 NS 2/6 non-N U-prot	0	6	0	1	5	5/5	6–11	NR	PR: 2–8 m, Relapse: 3 m
Naik.Sachin.2023. [48]	2	NS	0	2	0	0	2	2/2	#1: 18 m #2: 10 m	NR	NR
Sethi.Supreet.2020. [50]	6	Heavy NS	4	1	1	0	3	1/2	NR	≈6 m	NR
Shi.Guo-Qing.2025. [51]	5	NR	3	2	0	0	NR	NR	6–18 m	1–8 m	NR, remission within 12 m
Sridharan.Krita.2025. [52]	3	2 NS 1 N U-prot	3	0	0	0	3	2/3 (1 NR)	4–13 m	1–9 m	3–7 m
Teisseyre.Maxime.2021. [55]	4	3 heavy N U-prot, 1 N U-prot	0	4	0	0	4	4/4	12 m	6–12	3: ≈6 m
Wang.Jinmeng.2025. [59]	2	1 heavy N U-prot, 1 NS	1	1	0	1	1	1/1	12 m	#1: PR: 4 m #2: PR: 7 m CR by 12 m	#1: ≈6 m #2: PR ≈5 m CR NR
Wang.Linna.2026. [60]	9	N U-prot	1	6	2	0	7	4/7	3–14 m	NR	NR
Xu.Qiuyu.2024. [62]	1	Heavy NS	0	0	1	0	0	N.A.	≈5 m	NA	NA
Zhang.Yao.2024. [64]	1	NS	0	0	1	0	1	1/1	≈12 m	NA	NA
Zheng.Zhenfeng.2023. [66]	3	2 NS 1 N U-prot	0	3	0	0	3	3/3	7–10 m	7–10 m	NR
Zamora.JorgeIvan.2025. [63]	1	NS	0	0	1	0	NR	NR	Loss at F-U at 3 m		
Subtotal	72		19	42	11	2	52 *	40/49 *			
**Reports with aggregate cohort-level outcome data**											
Cheng.Xi.2025. [28]	30	NR in RTX group	14	12	4	2	NR in RTX group	NR in RTX group	NR in RTX group	Median 10 m	NR in RTX group
Dong.Rui.2025. [33]	11	NR in RTX group	2	9	0	NR	NR in RTX group	NR RTX group	≈12 m	NR	NR in RTX group
Li.Huxian.2026. [41]	17	NS	9	6	2	0	16	11/12	12.6 ±5 m	Median 4.4 m	NR in RTX group
Li.Luyao.2025. [42]	21	NR	NR in RTX group	NR in RTX group	NR in RTX group	0	NR	NR	≈12 m	≈9 m	NR in RTX group
Lin.Yuxin.2024. [45]	12	NR, only aggregate data	4	8	0	0	NR	NR	≈13.6 m In whole group	≈3.5 m In whole group	NR in RTX group
Podesta.ManuelAlfredo.2024. [49]	17	NS + middle impairment of KF	10 had	CR or PR	7	NR	14	7/14 ↓ 92.3% titers at 24 months	median 23.9 m	≈5 m	NR; improvement documented during follow-up (24 m)
Su.Wenyan.2025. [53]	20	NS	2	14	4	0	16	11/12	Median 9 m	Median 4.5 m	NR
Su.Xiaole.2024. [54]	14	5/14 NS 4/14 N U-prot 5/14 nonN U-prot	3	6	5	0	10	7/10	Median9.4 m	NR	NR
Teisseyre.Maxime.2025. [56]	15	NS or N U-prot	1	12	2	5	13	12/13	Median 8 m	≈5 m	NR in RTX group
Tian.Li.2026. [57]	25	NS or Heavy N U-prot with variable impairment of KF	4	16	5	2	21	13/21	6–30 m	NR	NR
Trillini.Matias.2026. [58]	20	NS with subnormal KF	5	12	3	0	9	9/9	At least 12 m	Median 9 m	NR
Zhao.Xiangling.2026. [65]	9	NR	7 had CR or PR		2	NR	NR	NR in RTX group	NR for RTX group		
Subtotal	211		NA	NA	34 *	9 *	99 *	70/90 *			

Footnotes: CR, complete remission; PR, partial remission; NR, not reported; NS, nephrotic syndrome; KF, kidney function; non-N U-prot, proteinuria in non-nephrotic range; N U-prot, proteinuria in nephrotic range; NA, not applicable. * Partially evaluated with the available data.

**Table 5:** Clinical outcomes after non-rituximab anti-CD20 in MCD.

Study-ID	Patients Assessed for Review (n)	Clinical Status Before Switching	Clinical CR (n)	Clinical PR (n)	No Clinical Remission (n)	Relapse (n)	Follow-Up	Time to First CR/PR	Duration of CR/PR
Angeletti.Andrea.2025. [25]	1	NS+ Normal KF	0	1	0	0	12 m	<3 m	≈9 m
Chen.Zewei.2025. [27]	3	2 non-N U-prot 1 N U-prot	3	0	0	1	6–12 m	≈1 m	5–11 m
Gauci.Pierre-Alexis.2025. [34]	1	For maintenance of CR	1	0	0	0	≈25 m	NA (already in CR; OBI used for maintenance)	12 m postpartum
Jin.Li.2024. [39]	1	non-N U-prot	1	0	0	0	9.9 m	≈1 m	≈8.9 m
LinYuxin.2025. [45]	8	non-N U-prot	8	0	0	0	12–25 m	NR	NR
Loeffler.Thomas.2025. [46]	1	Likely NS	1	0	0	0	36 m	≈1 m	36 m
Wang.Qiang.2024. [61]	1	NS	1	0	0	0	12 m	≈2 m	≈10 m
Zamora Jorge Ivan.2025. [63]	2	1 NS 1 non-N U-prot	0	2	0	0	12 m	≈6 m	≈6 m
Subtotal	18		15	3	0	1			

Footnotes: n, number; CR, complete remission; PR, partial remission; NS, nephrotic syndrome; KF, kidney function; non-N U-prot, proteinuria in non-nephrotic range; 1 N U-prot, proteinuria in nephrotic range; NA, not applicable.

**Table 6:** Clinical outcomes after non-rituximab anti-CD20 in FSGS.

Study-ID	Patients Assessed for Review (n)	Clinical Status Before Switching	Clinical CR (n)	Clinical PR (n)	No Clinical Remission (n)	Relapse (n)	Follow-Up	Time to First CR/PR	Duration of CR/PR
ChiliquingaMorales.Byron.2025. [30]	1	NS	0	0	1	0	11 m	NA	NA
Henriques.AndreiaRita.2026. [36]	1	non-N U-prot	1	0	0	0	11.5 m	2 m	≈9.5 m
LinYuxin.2025. [45]	3	non-N U-prot	3	0	0	2	18–26 m	NR	NR
Zamora Jorge Ivan.2025. [63]	1	NS	0	1	0	1	12 m	≈6 m	<6 m
Subtotal	6		4	1	1	3			

Footnotes: n, number; CR, complete remission; PR, partial remission; NS, nephrotic syndrome; KF, kidney function; non-N U-prot, proteinuria in non-nephrotic range; N U-prot, proteinuria in nephrotic range; NA, not applicable.

**Table 7:** Safety report of new generation anti-CD20 Ab.

Study-ID	Patients Assessed for Safety (n)	Any Reported Adverse Event (Yes/No/NR)	During Infusion (Yes/No)	Description	During Follow-Up (Yes/No/R)	Description
Angeletti.Andrea.2025. [25]	6 *	Yes	No	—	Yes	Impetigo
Boyer-Suavet.Sonia.2020. [26]	44 *	NR	NR		NR	
Chen.Zewei.2025. [27]	12 *	NR	NR		NR	
Cheng.Xi.2025. [28]	55 *	Yes	Yes	Chills, fever, digestive symptoms, dyspnea, palpitation	Yes	URTI; pneumonia, Herpes, UTI, soft tissue infection, death, abnormal liver function
Cheng.Xueyang.2025. [29]	74 *	NR	NR		NR	
ChiliquingaMorales.Byron.2025. [30]	1	NR	NR		NR	
deLaPradaAlvarez.FranciscoJose.2024. [31]	1	No	No	—	No	—
Ding.Qingyun.2025. [32]	3 *	Yes	Yes	Transient fever, chills	Yes	URTI
Dong.Rui.2025. [33]	15 *	Yes	No	—	Yes	Transient liver dysfunction; tuberculosis test conversion
Gauci.Pierre-Alexis.2025. [34]	3 *	No	No	No maternal or fetal adverse event	No	—
Guo.Jianying.2026. [35]	1	Yes	Yes	Chills, fever °C, tachycardia	NR	
Henriques.AndreiaRita.2026. [36]	1	Yes	No	—	Likely unrelated	Recurrent UTIs attributed to dapagliflozin, not obinutuzumab
Hudson.Rebecca.2022. [37]	2	No	No	—	No	—
Jha.VijoyKumar.2025. [38]	6	NR	NR		NR	
Jin.Li.2024. [39]	6 *	No	No	—	No	possible missed minor infections
Klomjit.Nattawat.2020. [40]	3	Yes	No	—	Yes	Localized herpes zoster
Li.Huxian.2026. [41]	17	Yes	Yes	Oropharyngeal discomfort, rash, or itching	Yes	COVID-19 infection requiring hospitalization
Li.Luyao.2025. [42]	25 *	Yes	Yes	Chills, fever, sweating with generalized discomfort	No	—
Li.Xue-Qi.2025. [43]	25 *	No	No	—	No	No hospitalized infections
Lin.Yuxin.2024. [44]	18 *	Yes	Yes	Pruritus *	Yes	COVID-19 pneumonia; pulmonary inflammation *
LinYuxin.2025. [45]	11	Yes	Yes	Itching, a sensation of throat tightness, drowsiness	Yes	COVID Infection; hypogammaglobulinemia
Liu.Yang.2026. [47]	6	Yes	Yes	Mild infusion reactions	No	—
Loeffler.Thomas.2025. [46]	1	No	No	—	No	—
Naik.Sachin.2023. [48]	2	Yes	No	—	Likely unrelated	Coronary artery disease (angioplasty), considered unrelated
Podesta.ManuelAlfredo.2024. [49]	17	Yes	Yes	Rash, edema, hypotension	Yes	Infection
Sethi.Supreet.2020. [50]	10 *	Yes	Yes	Wheezing	Yes	leukopenia; URTI, UTI
Shi.Guo-Qing.2025. [51]	75 *	Yes	Yes	Headache, digestive symptoms, rash, malaise in all cohort	Yes	Neutropenia
Sridharan.Krita.2025. [52]	3	No	No	—	No	—
Su.Wenyan.2025. [53]	20	Yes	Yes	Rash, nausea, tachycardia	Yes	Pneumonia
Su.Xiaole.2024. [54]	59 *	Yes	Yes	Mild infusion reactions	Yes	Neutropenia; bronchitis; UTI
Teisseyre.Maxime.2021. [55]	8 *	No	No	—	No	—
Teisseyre.Maxime.2025. [56]	34 *	Yes	Yes	Mild infusion reactions	Yes	UTI; digestive disorders; pain
Tian.Li.2026. [57]	25	Yes	Yes	Chills, skin rash, thoracic pain	No	—
Trillini.Matias.2026. [58]	20	Yes	Yes	Skin and facial rash, chills, rhinitis, bronchospasm	Yes	Delayed muscular and osteoarticular pain, asthenia, and elevated serum D-dimer
Wang.Jinmeng.2025. [59]	2	Yes	No	—	Yes	Penile herpes
Wang.Linna.2026. [60]	60 *	Yes	Yes	Rash, chills, Headache, dry throat	Yes	URI, Abnormal liver function, UTI, transient creatinine elevation
Wang.Qiang.2024. [61]	1	No	No	—	No	—
Xu.Qiuyu.2024. [62]	1	No	No	—	NR	No structured safety monitoring
Zamora Jorge Ivan.2025. [63]	6 *	Yes	Yes	Not specified mild infusion reactions;	Yes	Hypogammaglobulinemia; lymphopenia
Zhang.Yao.2024. [64]	1	No	No	—	No	—
Zhao.Xiangling.2026. [65]	31 *	Yes	yes	Fever, hypotension, tachycardia, and facial flushing	No	—
Zheng.Zhenfeng.2023. [66]	3 *	Yes	Yes	Fever; mild sore throat, rhinorrhea	Yes	mild URTI
Zhu.Shuhua.2026. [67]	1	No	No	—	No	—

Footnotes: n, number; NR, not reported/uncertain attribution; URTI, upper respiratory tract infection; URI, upper respiratory infection; UTI, urinary tract infection; * adverse events were not separable for the eligible subgroup.

**Table 8:** Certainty according to GRADE assessment.

Outcome	Risk of Bias	Inconsistency	Indirectness	Imprecision	Publication Bias	Certainty
MN: CR or PR	Serious	Serious	Not serious	Serious	Suspected	Very Low
MN: immunological remission	Serious	Serious	Not serious	Serious	Suspected	Very Low
MN: relapse	Serious	Not assessable	Not serious	Very serious	Strongly suspected	Very low
MCD: CR or PR	Serious	Not assessable	Serious	Very serious	Strongly suspected	Very low
FSGS: CR or PR	Serious	Serious/not assessable	Serious	Very serious	Strongly suspected	Very low
IgAN: CR or PR	Serious	Not assessable	Serious	Very serious	Strongly suspected	Very low
Adverse events	Serious	Not serious/not assessable	Not serious	Very serious	Strongly suspected	Very low

Footnotes: MN, membranous nephropathy; MCD, minimal change disease; FSGS, focal segmental glomerulosclerosis; IgAN, IgA nephropathy; CR, complete remission; PR, partial remission.

## Data Availability

All data extracted for this systematic review are reported in the article and its Supplementary Materials.

## Supplementary Materials

The following supporting information can be downloaded at: https://www.mdpi.com/article/10.3390/antib15050095/s1, Table S1: Patient-level and eligible-subgroup clinical and laboratory outcomes in membranous nephropathy; Table S2: Aggregate clinical and laboratory outcomes in membranous nephropathy cohort studies; Table S3: Patient-level clinical and laboratory outcomes in minimal change disease; Table S4: Patient-level clinical and laboratory outcomes in focal segmental glomerulosclerosis.

## Abbreviations

The following abbreviations are used in this manuscript:

*MN*	*Membranous nephropathy*
*MCD*	*Minimal change disease*
*FSGS*	*Focal segmental glomerulosclerosis*
*IgAN*	*IgA nephropathy*
*MPGN*	*Membranoproliferative glomerulonephritis*
*CR*	*Complete remission*
*PR*	*Partial remission*
*eGFR*	*Estimated glomerular filtration rate*
*UPCR*	*Urinary protein-to-creatinine ratio*
*NR*	*Not reported*
*CS*	*Corticosteroids*
*CNI*	*Calcineurin inhibitors*
*MMF*	*Mycophenolate mofetil*
*RTX*	*Rituximab*
*F-U*	*Follow-up*

## Appendix A

**Table A1:** Prisma Checklist.

Section and Topic	Item #	Checklist Item	Location Where Item Is Reported
**TITLE**			
Title	1	Identify the report as a systematic review.	Page 1
**ABSTRACT**			
Abstract	2	See the PRISMA 2020 for Abstracts checklist.	Page 1
**INTRODUCTION**			
Rationale	3	Describe the rationale for the review in the context of existing knowledge.	Reported—Introduction—opening five paragraphs.
Objectives	4	Provide an explicit statement of the objective(s) or question(s) the review addresses.	Reported—Introduction—final two paragraphs, including the within-patient review question and objectives.
**METHODS**			
Eligibility criteria	5	Specify the inclusion and exclusion criteria for the review and how studies were grouped for the syntheses.	Reported—Methods—Eligibility criteria; Outcome Definitions; Reasons for Switching; Data synthesis.
Information sources	6	Specify all databases, registers, websites, organizations, reference lists and other sources searched or consulted to identify studies. Specify the date when each source was last searched or consulted.	Reported—Methods—Electronic searches. Page 5
Search strategy	7	Present the full search strategies for all databases, registers and websites, including any filters and limits used.	Reported—Methods—Electronic searches. Full PubMed and Scopus strategies are shown. Page 6
Selection process	8	Specify the methods used to decide whether a study met the inclusion criteria of the review, including how many reviewers screened each record and each report retrieved, whether they worked independently, and if applicable, details of automation tools used in the process.	Reported—Methods—Study selection; Results opening paragraph; Figure 1.
Data collection process	9	Specify the methods used to collect data from reports, including how many reviewers collected data from each report, whether they worked independently, any processes for obtaining or confirming data from study investigators, and if applicable, details of automation tools used in the process.	Reported—Methods—Data Collection.
Data items	10a	List and define all outcomes for which data were sought. Specify whether all results that were compatible with each outcome domain in each study were sought (e.g., for all measures, time points, analyses), and if not, the methods used to decide which results to collect.	Reported—Methods—Outcomes; Outcome Definitions; Table 1.
	10b	List and define all other variables for which data were sought (e.g., participant and intervention characteristics, funding sources). Describe any assumptions made about any missing or unclear information.	Reported—Methods—Data Collection
Study risk of bias assessment	11	Specify the methods used to assess risk of bias in the included studies, including details of the tool(s) used, how many reviewers assessed each study and whether they worked independently, and if applicable, details of automation tools used in the process.	Reported—Methods—Quality and relevance assessment; Results Section 3.5; Appendix B.
Effect measures	12	Specify for each outcome the effect measure(s) (e.g., risk ratio, mean difference) used in the synthesis or presentation of results.	Reported—Partial—Results are presented as counts, proportions and descriptive continuous data.
Synthesis methods	13a	Describe the processes used to decide which studies were eligible for each synthesis (e.g., tabulating the study intervention characteristics and comparing against the planned groups for each synthesis (item #5)).	Reported—Methods—Data synthesis; studies grouped by disease, anti-CD20 agent, switch reason, design and reporting level.
	13b	Describe any methods required to prepare the data for presentation or synthesis, such as handling of missing summary statistics, or data conversions.	Reported—Methods—Data Collection; Outcome Definitions; Identification and Management of Overlapping Reports.
	13c	Describe any methods used to tabulate or visually display results of individual studies and syntheses.	Reported—Methods—Data synthesis; Figure 1; Table 1, Table 2, Table 3, Table 4, Table 5, Table 6, Table 7 and Table 8 and Tables S1–S4 and Appendix B.
	13d	Describe any methods used to synthesize results and provide a rationale for the choice(s). If meta-analysis was performed, describe the model(s), method(s) to identify the presence and extent of statistical heterogeneity, and software package(s) used.	Reported—Methods—Assessment of heterogeneity and Data synthesis. Narrative synthesis was selected because clinical and methodological heterogeneity precluded meta-analysis.
	13e	Describe any methods used to explore possible causes of heterogeneity among study results (e.g., subgroup analysis, meta-regression).	Reported—Methods—Assessment of heterogeneity; descriptive exploration by disease, agent, regimen, switch reason, outcome definition and follow-up.
	13f	Describe any sensitivity analyses conducted to assess robustness of the synthesized results.	Not applicable—Not applicable—no sensitivity analysis was performed. State this explicitly in Methods.
Reporting bias assessment	14	Describe any methods used to assess risk of bias due to missing results in a synthesis (arising from reporting biases).	Reported Certainty assessment paragraph.
Certainty assessment	15	Describe any methods used to assess certainty (or confidence) in the body of evidence for an outcome.	Reported—Methods—Certainty assessment (GRADE).
**RESULTS**			
Study selection	16a	Describe the results of the search and selection process, from the number of records identified in the search to the number of studies included in the review, ideally using a flow diagram.	Reported—Results—opening paragraph and Figure 1.
	16b	Cite studies that might appear to meet the inclusion criteria, but which were excluded, and explain why they were excluded.	Reported Figure 1.
Study characteristics	17	Cite each included study and present its characteristics.	Reported—Results Section 3.1; Table 2.
Risk of bias in studies	18	Present assessments of risk of bias for each included study.	Reported—Results Section 3.5; Appendix B.
Results of individual studies	19	For all outcomes, present, for each study: (a) summary statistics for each group (where appropriate) and (b) an effect estimates and its precision (e.g., confidence/credible interval), ideally using structured tables or plots.	Reported—Results Section 3.3 and Section 3.4; Table 4, Table 5, Table 6 and Table 7.
Results of syntheses	20a	For each synthesis, briefly summarize the characteristics and risk of bias among contributing studies.	Reported—Results Section 3.3, Section 3.4 and Section 3.5 and Discussion—syntheses are presented by glomerular disease and interpreted in light of study design and risk of bias.
	20b	Present results of all statistical syntheses conducted. If meta-analysis was done, present for each the summary estimate and its precision (e.g., confidence/credible interval) and measures of statistical heterogeneity. If comparing groups, describe the direction of the effect.	Not applicable—no meta-analysis or other statistical synthesis was performed.
	20c	Present results of all investigations of possible causes of heterogeneity among study results.	Reported—Results—descriptive findings stratified by disease and reason for switching.
	20d	Present results of all sensitivity analyses conducted to assess the robustness of the synthesized results.	Not applicable—no sensitivity analysis was conducted.
Reporting biases	21	Present assessments of risk of bias due to missing results (arising from reporting biases) for each synthesis assessed.	Reported—Results/Certainty and Discussion.
Certainty of evidence	22	Present assessments of certainty (or confidence) in the body of evidence for each outcome assessed.	Reported—Results—Certainty subsection; Table 8.
**DISCUSSION**			
Discussion	23a	Provide a general interpretation of the results in the context of other evidence.	Reported—Discussion—overall interpretation and comparison with previous systematic reviews.
	23b	Discuss any limitations of the evidence included in the review.	Reported—Discussion—limitations of the available evidence.
	23c	Discuss any limitations of the review processes used.	Reported—limitation paragraph discussion.
	23d	Discuss implications of the results for practice, policy, and future research.	Reported—Discussion—clinical implications, future research paragraph and Conclusions.
**OTHER INFORMATION**			
Registration and protocol	24a	Provide registration information for the review, including register name and registration number, or state that the review was not registered.	Reported—Methods—first paragraph: Open Science Framework registration, OSF psdk6.
	24b	Indicate where the review protocol can be accessed, or state that a protocol was not prepared.	Reported—Methods—first paragraph: protocol accessible through OSF psdk6.
	24c	Describe and explain any amendments to information provided at registration or in the protocol.	No amendments were made to the registered protocol.
Support	25	Describe sources of financial or non-financial support for the review, and the role of the funders or sponsors in the review.	Reported—Funding statement—no external funding.
Competing interests	26	Declare any competing interests of review authors.	Reported—Conflicts of Interest statement—authors declare no conflicts of interest.
Availability of data, code and other materials	27	Report which of the following are publicly available and where they can be found: template data collection forms; data extracted from included studies; data used for all analyses; analytic code; any other materials used in the review.	Reported—Methods paragraph.

## Appendix B

**Table A2:** Quality assessment for case report according to JBI CHECKLIST.

Study-ID	Q1	Q2	Q3	Q4	Q5	Q6	Q7	Q8	Overall Appraisal
ChiliquingaMorales.Byron.2025. [30]	yes	yes	yes	yes	yes	yes	unclear	Yes	Well-documented case report showing partial response after rituximab failure, but concomitant tacrolimus, diagnostic evolution, absent safety reporting, and single-patient design provide very low-certainty evidence.
deLaPradaAlvarez.FranciscoJose.2024. [31]	yes	yes	yes	yes	yes	yes	yes	Yes	Well-documented case report, but concomitant tacrolimus, diagnostic evolution, absent safety reporting, and single-patient design provide very low-certainty evidence.
Guo.Jianying.2026. [35]	yes	yes	yes	yes	yes	yes	yes	Yes	Well-documented case report, but delayed effects of prior rituximab, natural disease course, supportive therapy, not clearly reported supportive therapy after obinutuzumab and safety surveillance was not described systematically.
Henriques.AndreiaRita.2026. [36]	yes	yes	yes	yes	yes	yes	yes	Yes	Detailed case report with objective outcomes and adequate follow-up, but concomitant immunosuppression, pre-emptive retreatment, and absent anti-rituximab testing limit causal certainty and generalizability.
Hudson.Rebecca.2022. [37]	yes	yes	yes	yes	yes	yes	yes	Yes	Detailed two-patient report with objective longitudinal outcomes, but extensive prior therapies.
Loeffler.Thomas.2025. [46]	yes	yes	yes	yes	yes	yes	yes	Yes	Detailed case report with long follow-up, but treatment during early remission, major concomitant therapies not clearly reported, uncertain adverse-event attribution.
Naik.Sachin.2023. [48]	yes	no	yes	yes	yes	yes	no	Yes	two-case report with objective outcomes, but highly selected patients, incomplete prior-treatment and safety data, safety surveillance was not described systematically.
Wang.Jinmeng.2025. [60]	yes	yes	yes	yes	yes	no	yes	Yes	Detailed timelines, biopsy confirmation and objective outcomes support reporting quality, but two selected cases, extensive previous therapy, incomplete follow-up and uncertain treatment attribution preclude causal or generalizable conclusions.
Wang.Qiang.2024. [61]	yes	yes	yes	yes	no	yes	unclear	Yes	The timeline is detailed, but tabulated final laboratory data and systematic safety reporting are incomplete.
Xu.Qiuyu.2024. [62]	yes	yes	yes	yes	no	yes	unclear	Yes	The pretreatment course is detailed, but post-obinutuzumab and safety reporting are sparse, and efficacy is emphasized despite no conventional CR or PR.
Zhang.Yao.2024. [64]	yes	no	yes	yes	no	no	yes	Yes	Clinical and laboratory improvement is described, but final proteinuria, albumin and creatinine differ between the narrative and Figure 1, and no B-cell data are available. Absence of recurrent hypokalemia with obinutuzumab is also reported, although broader safety monitoring is limited.
Zhu.Shuhua.2026. [67]	yes	yes	yes	yes	yes	yes	unclear	Yes	Outcome reporting is detailed, but safety surveillance and infusion details are incomplete, and response timing is described as both 2 weeks and 1 month.

**Table A3:** Quality assessment for case series according to JBI CHECKLIST.

Study-ID	Q1	Q2	Q3	Q4	Q5	Q6	Q7	Q8	Q9	Q10	Overall Appraisal
Angeletti.Andrea.2025. [25]	unclear	unclear	unclear	unclear	unclear	yes	unclear	yes	no	Yes	Small uncontrolled case series suggesting obinutuzumab efficacy after rituximab failure, but limited by unclear patient selection, incomplete baseline reporting, no comparator, and very low certainty
Chen.Zewei.2025. [27]	yes	yes	yes	yes	unclear	yes	yes	yes	yes	No	Consecutive retrospective case series with clear outcomes, but very small heterogeneous sample, variable dosing and follow-up, no comparator, and inclusion of two minors limit certainty and generalizability
Gauci.Pierre-Alexis.2025. [34]	yes		yes	yes	no	yes	yes	yes		Yes	Well-described single obinutuzumab pregnancy case, but preconception exposure, prior rituximab-induced remission, absent comparator, limited laboratory detail
Ding.Qingyun.2025. [32]	unclear	yes	yes	unclear	unclear	yes	yes	yes	no	N.A.	Well-described three-patient case series with objective outcomes, but unclear selection, heterogeneous dosing, concomitant therapies, no comparator, and only one rituximab-exposed patient provide very low-certainty evidence
Jha.VijoyKumar.2025. [38]	no	yes	yes	unclear	unclear	yes	yes	yes	no	N.A.	case series with detailed individual outcomes, but unclear selection, heterogeneous prior therapies, no standardized follow-up, incomplete safety reporting
Jin.Li.2024. [39]	unclear	yes	yes	unclear	unclear	yes	yes	yes	no	N.A.	clearly reported outcomes, but unclear selection, concomitant corticosteroids, short follow-up, incomplete safety capture
Klomjit.Nattawat.2020. [40]	yes	yes	yes	unclear	unclear	yes	yes	yes	no	N.A.	Objective longitudinal outcomes, but unclear selection, heterogeneous prior therapies, and limited safety data provide
Lin.Yuxin.2025. [45]	yes	yes	yes	unclear	unclear	yes	no	yes	yes	No	Retrospective case series with detailed longitudinal outcomes, but heterogeneous baseline status, concomitant immunosuppression, unclear consecutive inclusion, data presented partial aggregate
Lin.Yuxin.2024. [44]	yes	yes	yes	unclear	unclear	yes	no	no	yes	No	retrospective case series with standardized outcomes, but unclear complete inclusion, heterogeneous prior treatments, small subgroups, and uncontrolled confounding limit causal certainty
Liu.Yang.2026. [47]	yes	yes	yes	unclear	yes	yes	yes	yes	yes	Yes	A small single-center retrospective case series with clear eligibility and outcome definitions, but limited by uncertain consecutive recruitment, potential selection bias, and short follow-up
Sethi.Supreet.2020. [50]	no	no	no	unclear	unclear	yes	no	yes	yes	No	Retrospective heterogeneous case series with objective outcomes, but unclear selection, mixed native and transplant disease, incomplete treatment details, no comparator, and limited safety ascertainment
Sridharan.Krita.2025. [52]	yes	yes	yes	yes	yes	yes	yes	no	yes	Yes	Retrospective heterogeneous case series with objective outcomes, clear selection, but some inconsistence between text and table
Teisseyre.Maxime.2022. [55]	yes	yes	yes	unclear	uncleasr	yes	no	yes	yes	Yes	Detailed longitudinal biomarker and treatment data strengthen reporting, but non-consecutive selection, tiny heterogeneous treatment groups, biomarker-guided allocation. Likely 2 duplicate cases
Zhao.Xiangling.2026. [65]	yes	yes	yes	unclear	unclear	yes	yes	yes	yes	Yes	Single-center case series with standardized outcomes, but uncertain consecutive and complete inclusion, complete-case selection, subgroup inconsistencies, and no comparator limit causal interpretation. Poorly description of previous rituximab subgroup
Zheng.Zhenfeng.2023. [66]	no	yes	yes	unclear	unclear	yes	no	no	yes	N.A.	Substantial missing outcome and treatment details, short variable follow-up, and inconsistencies in age and serologic time points
Zamora Jorge Ivan.2025 [63]	yes	yes	yes	unclear	unclear	yes	yes	no	yes	Yes	Biopsy confirmation and patient-level laboratory data strengthen reporting, but selective inclusion, heterogeneous diseases and drugs, undefined remission criteria, one loss to follow-up, and tiny sample severely limit inference

N.A., Not Applicable.

**Table A4:** Quality assessment for observational studies according to JBI CHECKLIST.

Study-ID	Q1	Q2	Q3	Q4	Q5	Q6	Q7	Q8	Q9	Q10	Q11	Overall appraisal
Boyer-Suavet.Sonia.2020. [26]	yes	yes	yes	unclear	no	unclear	yes	yes	unclear	no	no	Retrospective single-center cohort with standardized measurements and adequate follow-up, but small sample, limited confounding control, post-baseline exposure classification, and highly uncertain ofatumumab evidence
Cheng.Xi.2025. [28]	no	yes	yes	yes	no	yes	yes	yes	no	no	yes	Retrospective single-center cohort with standardized outcomes and multivariable analysis, but baseline imbalance, excluded losses to follow-up, treatment heterogeneity, and no direct rituximab comparator limit certainty
Cheng.Xueyang.2025. [29]	no	yes	yes	no	no	yes	yes	no	no	no	yes	Retrospective cohort with standardized assays and defined outcomes, but substantial baseline imbalance, incomplete follow-up, limited confounding control, inconsistent reporting, and very low-certainty obinutuzumab subgroup evidence
Dong.Rui.2025. [33]	N.A.	unclear	yes	no	no	unclear	yes	unclear	unclear	no	no	Retrospective single-center cohort with objective aggregate outcomes, but no concurrent comparator, incomplete rituximab exposure data, uncontrolled confounding, uncertain follow-up completeness
Li.Huxian.2025. [41]	N.A.	N.A.	yes	unclear	yes	yes	yes	yes	yes	N.A.	unclear	This small retrospective single-arm cohort provides objective within-patient evidence of response, but the absence of a concurrent comparator, variable dosing, incomplete biomarker data, and residual confounding substantially limit causal certainty
Li.Luyao.2025. [42]	no	no	yes	no	no	yes	yes	yes	yes	N.A.	no	Retrospective two-center cohort with consecutive enrollment and standardized outcomes, but variable dosing, concomitant immunosuppression, no comparator, limited confounding control, and non-separable rituximab subgroup reduce certainty
Li.Xue-Qi.2025. [43]	no	yes	yes	unclear	no	yes	yes	no	yes	N.A.	no	Retrospective comparative cohort with standardized outcomes and a concurrent rituximab group, but baseline imbalance, crossover, short follow-up, and limited confounding adjustment reduce certainty
Podesta.ManuelAlfredo.2023. [49]	yes	yes	yes	unclear	yes	yes	yes	yes	yes	yes	yes	retrospective case series with standardized assessment and complete screening, but small heterogeneous subgroups, possible residual confounding
Shi.Guo-Qing.2025. [51]	N.A.	N.A.	yes	unclear	No	yes	yes	yes	unclear	N.A.	unclear	This retrospective single-center cohort provides objective short-term outcome data, but inadequate confounding control, and selective long-term follow-up substantially limit causal inference. Furthermore only 5 cases were adequate for our purpose
Su.Wenyan.2025. [53]	N.A.	N.A.	yes	yes	no	Yes	yes	no	unclear	no	no	Well-defined cohort and objective outcomes support credibility, but retrospective single-center design, small sample, follow-up-based selection, and underpowered exploratory analyses substantially limit causal inference
Su.Xiaole.2024. [54]	no	yes	yes	yes	yes	yes	yes	yes	no	no	yes	Objective outcomes and adjusted analyses strengthen the study, but retrospective single-center design, baseline imbalance, follow-up-based exclusions, absent parallel control, and residual confounding limit causal inference
Teisseyre.Maxime.2025. [56]	yes	yes	yes	yes	no	yes	no	yes	yes	N.A.	no	Multicenter recruitment and objective outcomes strengthen the study, but treatment allocation depends on nephrology center, pooled drugs, small groups, unequal dosing, center effects, and unadjusted confounding substantially weaken comparative conclusion
Tian.Li.2026. [57]	N.A.	N.A.	yes	yes	no	yes	yes	yes	unclear	no	no	Consecutive inclusion, biopsy confirmation and objective longitudinal outcomes strengthen the study, but uncontrolled retrospective design, variable dosing, incomplete follow-up clarification, small nonresponder group and unadjusted confounding limit inference
Wang.Linna.2026. [60]	no	yes	yes	yes	no	yes	yes	yes	no	no	no	This retrospective cohort provides useful preliminary data, but substantial baseline imbalance, unadjusted analyses, incomplete follow-up, and inconsistent reporting confer a moderate-to-high risk of bias

N.A., Not Applicable.

**Table A5:** Quality assessment for Prospective (quasi-experimental) study according to JBI CHECKLIST.

Study-ID	Q1	Q2	Q3	Q4	Q5	Q6	Q7	Q8	Q9	Overall Appraisal
Trillini.Matias.2026. [58]	yes	N.A.	N.A.	no	yes	yes	yes	yes	yes	Prospective design, consecutive enrolment, complete follow-up and objective repeated outcomes support credibility, but the uncontrolled single-arm design, small sample and historical assumptions preclude causal or comparative conclusions

N.A., Not Applicable.

## References

[1] Yandian F., Sethi S., Fervenza F.C., Caravaca-Fontán F. (2025). Targeting CD20 for B-cell Depletion in Autoimmune Kidney Disease: Next Generation. BioDrugs.

[2] Gopal A.K., Press O.W. (1999). Clinical applications of anti-CD20 antibodies. J. Lab. Clin. Med..

[3] Santos J.E., Fiel D., Santos R., Vicente R., Aguiar R., Santos I., Amoedo M., Pires C. (2020). Rituximab use in adult glomerulopathies and its rationale. J. Bras. Nefrol..

[4] Leget G.A., Czuczman M.S. (1998). Use of rituximab, the new FDA-approved antibody. Curr. Opin. Oncol..

[5] Onrust S.V., Lamb H.M., Balfour J.A. (1999). Rituximab. Drugs.

[6] Schilder R. (1999). Rituximab immunotherapy. Cancer Biother. Radiopharm..

[7] Zhang J., Bian L., Ma F.Z., Jia Y., Lin P. (2018). Efficacy and safety of rituximab therapy for membranous nephropathy: A meta-analysis. Eur. Rev. Med. Pharmacol. Sci..

[8] Zhang Y., Chen H., Zhang H., Huang Y. (2025). Evaluating rituximab against cyclophosphamide or cyclosporine in idiopathic membranous nephropathy: A meta-analysis. Eur. J. Med. Res..

[9] Huang L., Dong Q.R., Zhao Y.J., Hu G.C. (2021). Rituximab for the management of idiopathic membranous nephropathy: A meta-analysis. Int. Urol. Nephrol..

[10] Hansrivijit P., Cheungpasitporn W., Thongprayoon C., Ghahramani N. (2020). Rituximab therapy for focal segmental glomerulosclerosis and minimal change disease in adults: A systematic review and meta-analysis. BMC Nephrol..

[11] Li J., Nie Z., Li G., Bao B. (2025). Efficacy and safety of rituximab in primary IgA nephropathy: A retrospective study. Clin. Exp. Nephrol..

[12] Cheng Y., Fu Q., Cui X., Hu M., Chang M., Jiang Y., Wang H. (2026). The successful use of rituximab in children with IgA nephropathy and minimal change-like lesions: A case series. Ren. Fail..

[13] Sun M., Wang L., Liu X., Xiao F., Dai H. (2024). The successful use of rituximab in IgA nephropathy patients with podocytopathy: A case series. Clin. Kidney J..

[14] Chiarenza D.S., Verrina E.E., Bigatti C., Caridi G., Spennacchio A., Kajana X., De Bortoli M., Cravedi P., Angeletti A. (2026). B-Cell Depletion in Glomerular Diseases: Not Always as Safe as We May Think. Kidney Med..

[15] Hartinger J.M., Kratky V., Hruskova Z., Slanar O., Tesar V. (2022). Implications of rituximab pharmacokinetic and pharmacodynamic alterations in various immune-mediated glomerulopathies and potential anti-CD20 therapy alternatives. Front. Immunol..

[16] Cartron G., Dacheux L., Salles G., Solal-Celigny P., Bardos P., Colombat P., Watier H. (2002). Therapeutic activity of humanized anti-CD20 monoclonal antibody and polymorphism in IgG Fc receptor FcgammaRIIIa gene. Blood.

[17] Kaegi C., Wuest B., Crowley C., Boyman O. (2021). Systematic Review of Safety and Efficacy of Second- and Third-Generation CD20-Targeting Biologics in Treating Immune-Mediated Disorders. Front. Immunol..

[18] Lurie A., Daneii P., Khan S., Khan Z., Mansuri H., Bizanti A. (2026). Obinutuzumab Versus Rituximab for the Treatment of Primary Membranous Nephropathy: A Systematic Review and Meta-Analysis. Life.

[19] Atay I., Eroz E., Korucu B., Oktan M.A., Bildaci Y.D., Deger S.M., Cavdar C., Heybeli C. (2026). Obinutuzumab for membranous nephropathy: A systematic review using pooled case-level extraction analysis. Acta Clin. Belg..

[20] Page M.J., McKenzie J.E., Bossuyt P.M., Boutron I., Hoffmann T.C., Mulrow C.D., Shamseer L., Tetzlaff J.M., Akl E.A., Brennan S.E. (2021). The PRISMA 2020 statement: An updated guideline for reporting systematic reviews. BMJ.

[21] (2021). KDIGO 2021 Clinical Practice Guideline for the Management of Glomerular Diseases. Kidney Int..

[22] Rovin B.H., Barratt J., Cook H.T., Noronha I.L., Reich H.N., Suzuki Y., Tang S.C., Trimarch H., Floege J. (2025). KDIGO 2025 Clinical Practice Guideline for the Management of Immunoglobulin A Nephropathy (IgAN) and Immunoglobulin A Vasculitis (IgAV). Kidney Int..

[23] Moola S., Munn Z., Tufanaru C., Aromataris E., Sears K., Sfetcu R., Currie M., Qureshi R., Mattis P., Mu P., Aromataris E.M.Z. (2020). Chapter 7: Systematic reviews of etiology and risk. JBI Manual for Evidence Synthesis.

[24] Alper B.S., Oettgen P., Kunnamo I., Iorio A., Ansari M.T., Murad M.H., Meerpohl J.J., Qaseem A., Hultcrantz M., Schünemann H.J. (2019). Defining certainty of net benefit: A GRADE concept paper. BMJ Open.

[25] Angeletti A., Caridi G., Kajana X., Bigatti C., Spennacchio A., Chiarenza D.S., Mortari G., Cravedi P., Verrina E.E. (2025). Obinutuzumab in Rituximab-Resistant Podocytopathies: A Single-Center Experience. Kidney Int. Rep..

[26] Boyer-Suavet S., Andreani M., Lateb M., Savenkoff B., Brglez V., Benzaken S., Bernard G., Nachman P.H., Esnault V., Seitz-Polski B. (2019). Neutralizing Anti-Rituximab Antibodies and Relapse in Membranous Nephropathy Treated with Rituximab. Front. Immunol..

[27] Chen Z., Xu D., Wu S., Liu W., Wu J., Yu S., Dai B., Mao Z., Gao X. (2025). The role of obinutuzumab in rituximab-refractory membranous nephropathy and minimal change disease. Clin. Kidney J..

[28] Cheng X., Zheng Z.-F., Qi Y., Cao X., Gao X.-Q., Fang Y.-X., Wei L., Shang W.-Y., Yan T.-K., Jia J.-Y. (2025). Efficacy and safety of obinutuzumab in primary membranous nephropathy: A real-world retrospective study. Front. Immunol..

[29] Cheng X., Zhou M., Chen C., Xue J., Liu B., Zhang Z., Zhang X., Zhou L., Cai T., Huang B. (2025). Clinical study of PLA2R epitope spreading for predicting proteinuria remission in primary membranous nephropathy. Front. Immunol..

[30] Morales B.C., Garrote P.S., Gómez A.R., Varas C.M., Gutiérrez P.G., González A.S., García L.C., Alonso C.S., Martínez C.M., de la Rosa N.L. (2025). Obinutuzumab in focal segmental glomerulonephritis resistant to treatment. Nefrologia (Engl. Ed.).

[31] de la Prada Álvarez F.J., Cintra Cabrera M., Almenara Tejederas M., Burgos Martin J., Alonso García F., Salgueira Lazo M. (2024). Obinutuzumab in the treatment of PLA2R-positive membranous glomerulonephritis resistant to treatment. Nefrologia (Engl. Ed.).

[32] Ding Q., Xue C., Gao X., Tang X., Zhang S., Liu Y., Dai B. (2025). Efficacy and safety of obinutuzumab on progressive IgA nephropathy: A case series. J. Nephrol..

[33] Dong R., Guo M.N., Sun J., Li S.X., Bian Q., Xu J. (2025). Obinutuzumab in treating refractory primary membranous nephropathy: A single-center study. Acad. J. Nav. Med. Univ..

[34] Gauci P.-A., Cremoni M., Delotte J., Vocila F., Esnault V.L.M., Seitz-Polski B., Teisseyre M. (2025). Treatment of nephrotic syndrome with anti-CD20 therapies in pregnancy: A case series and review of the literature. Ren. Fail..

[35] Guo J., Zhong A., Wu D., Liu Y., Chen Y., Xu Y., Xu R., Wan Q. (2026). Obinutuzumab after anti-rituximab antibody-associated treatment failure in membranous nephropathy: A case report. Front. Immunol..

[36] Henriques A.R., Venda J., Ferreira E., Oliveira N., Sá H. (2026). Successful use of obinutuzumab in focal segmental glomerulosclerosis with inadequate response to rituximab: A case report. Front. Nephrol..

[37] Hudson R., Rawlings C., Mon S.Y., Jefferis J., John G.T. (2022). Treatment resistant M-type phospholipase A2 receptor associated membranous nephropathy responds to obinutuzumab: A report of two cases. BMC Nephrol..

[38] Jha V.K., Panda S.K., Rai S.K., Srivastava A.K. (2025). Obinutuzumab in Difficult to Treat Phospholipase A2 Receptor Positive Membranous Nephropathy: Our Experience at a Tertiary Care Center in North India. Indian J. Nephrol..

[39] Jin L., Liu X., Li H., Dang X., Wang Z., Niu D., Zhang X., Sun J., Hao D., Lu W. (2024). Obinutuzumab is effective for the treatment of frequently-relapsing/steroid-dependent minimal change disease in adults. Nephrol. Dial. Transplant..

[40] Klomjit N., Fervenza F.C., Zand L. (2020). Successful Treatment of Patients with Refractory PLA(2)R-Associated Membranous Nephropathy with Obinutuzumab: A Report of 3 Cases. Am. J. Kidney Dis..

[41] Li H., Jin L., Xie X., Sun J., Niu D., Feng J., Xu G., Zhang X., Khalaf A.M.S., Lu W. (2026). Additive Obinutuzumab Achieves High Remission Rates in Rituximab-Refractory Membranous Nephropathy. Am. J. Nephrol..

[42] Li L., Wang S., Wei M., Zhang Q., Fang L., Fan X., Cheng M., Zhang C., Liang H., Xiao X. (2025). Observation of the efficacy and safety of obinutuzumab in the treatment of refractory idiopathic membranous nephropathy. Int. Urol. Nephrol..

[43] Li X.Q., Liu Y., Cai Z.Y., Lv T.G., Hao J. (2025). Obinutuzumab is Effective as an Initial Treatment for PLA2R-Associated Primary Membranous Nephropathy: A Retrospective, Single-Center Trial. Drug Des. Devel Ther..

[44] Lin Y., Han Q., Chen L., Wang Y., Ren P., Liu G., Lan L., Lei X., Chen J., Han F. (2024). Obinutuzumab in Refractory Membranous Nephropathy: A Case Series. Kidney Med..

[45] Lin Y., Pan Y., Han Q., Xu J., Wang J., Lei X., Chen L., Wang Y., Ren P., Lan L. (2025). Obinutuzumab May Be an Effective and Safe Option for Adult Minimal Change Disease and Focal Segmental Glomerulosclerosis Patients after Multitarget Therapy Including Rituximab. Am. J. Nephrol..

[46] Loeffler T., Flahault A., Alla A., Frimat L., Kormann R. (2025). Obinutuzumab, an Anti-CD20, in Refractory Adult Autoimmune Podocytopathies: Report of 2 Cases. Kidney Med..

[47] Liu Y., Wu D., Xue R., Chen Y., Wan Q. (2026). Obinutuzumab as a Rescue Therapy in Anti-Rituximab Antibody-Positive Primary Membranous Nephropathy. Kidney Int. Rep..

[48] Naik S., Shukla S., Av N., Kumar V., Sekar A., Nada R., Rathi M., Kohli H.S., Ramachandran R. (2023). Obinutuzumab in Refractory Phospholipase A2 receptor-Associated Membranous Nephropathy with Severe CKD. Kidney Int. Rep..

[49] Podestà M.A., Trillini M., Portalupi V., Gennarini A., Tomatis F., Villa A., Perna A., Rubis N., Remuzzi G., Ruggenenti P. (2024). Ofatumumab in Rituximab-Resistant and Rituximab-Intolerant Patients with Primary Membranous Nephropathy: A Case Series. Am. J. Kidney Dis..

[50] Sethi S., Kumar S., Lim K., Jordan S.C. (2020). Obinutuzumab is Effective for the Treatment of Refractory Membranous Nephropathy. Kidney Int. Rep..

[51] Shi G.Q., Yu R., Wu Y., Zhou H., Zhao Z.X., Zheng X.M., Wang Y.Q., Zhang B.R., Zhang Y.Z. (2025). Obinutuzumab for PLA2R-associated membranous nephropathy. Nephrol. Dial. Transplant..

[52] Sridharan K., Gopal B., Wilson S., Pham A., Hutton H. (2025). Obinutuzumab in Rituximab-resistant and recurrent membranous nephropathy: A case-series. J. Nephrol..

[53] Su W., Li J., Men J., Zhong S., Wang Y., Zhang Y., Xu L., Gao J., Liu D., Wang R. (2025). Obinutuzumab is effective for the treatment of rituximab-refractory PLA2R-associated membranous nephropathy. Clin. Kidney J..

[54] Su X., Wu B., Tie X., Guo X., Feng R., Qiao X., Wang L. (2024). Obinutuzumab as Initial or Second-Line Therapy in Patients with Primary Membranous Nephropathy. Kidney Int. Rep..

[55] Teisseyre M., Boyer-Suavet S., Crémoni M., Brglez V., Esnault V., Seitz-Polski B. (2021). Analysis and Management of Rituximab Resistance in PLA2R1-Associated Membranous Nephropathy. Kidney Int. Rep..

[56] Teisseyre M., Allinovi M., Audard V., Cremoni M., Belvederi G., Karamé A., Accinno M., Duquesne J., Sharma V., Fernandez C. (2025). Obinutuzumab and Ofatumumab are More Effective Than Rituximab in the Treatment of Membranous Nephropathy Patients with Anti-Rituximab Antibodies. Kidney Int. Rep..

[57] Tian L., Pei H., Dong T., Zhou M., Li S., Zhao X., Liang X., Xing L. (2026). Efficacy and safety of obinutuzumab as rescue therapy for rituximab-resistant and rituximab-recurrent primary membranous nephropathy: A retrospective cohort study. Open Med..

[58] Trillini M., Portalupi V., Gennarini A., Surdi L., Currado A., Marasà M., Podestà M.A., Villa A., Peracchi T., Fidone D. (2026). Prospective Cohort Study of Obinutuzumab in Rituximab-Resistant Primary Membranous Nephropathy. Kidney Int. Rep..

[59] Wang J., Wu W., Wu H. (2025). Obinutuzumab for treatment of membranous nephropathy in patients positive and negative for the phospholipase A(2) receptor antibody: Case reports. Front. Immunol..

[60] Wang L., Yang M., Yan L., Shao F. (2026). First-line and Second-line Therapy of Zuberitamab in Idiopathic Membranous Nephropathy: A Single-center Retrospective Study. Kidney Med..

[61] Wang Q., Lin L., Zhen J., Jiang B., Liu G. (2024). Case report: Effective treatment of rituximab-resistant minimal change disease with obinutuzumab in an adult. Front. Immunol..

[62] Xu Q.Y., Chen G., Yang C.H., Zheng K., Ma J., Li C., Fan X.H., Ye W., Wen Y.B., Chen L.M. (2024). The 508th case: Recurrent edema of bilateral lower extremities with proteinuria. Zhonghua Nei Ke Za Zhi.

[63] Zamora J.I., López-Martínez M., Ramos N., Bermejo S., Agraz I., García-Carro C., Patricio M., León J.C., Toapanta N., Bestard O. (2025). Experience with new anti-CD20 monoclonal antibodies for immune-mediated glomerulopathies in a tertiary hospital. Nefrologia (Engl. Ed.).

[64] Zhang Y., Sun J., Gao J., Sun W., Xu L., Zhai C., Su W., Wang H. (2024). Case report: One case of refractory membranous nephropathy with hypokalemia after rituximab infusion was switched to obinutuzumab without recurrence of hypokalemia. Front. Pharmacol..

[65] Zhao X., Chang X., Wang Y., Chen D., Yu M., Zhao J., Zhu H., Mao Y., Juan C. (2026). Obinutuzumab in the treatment of idiopathic refractory membranous nephropathy: An observational case series. Front. Med..

[66] Zheng Z., Cheng X., Qi Y., Shang W., Wei L., Li D., Jia J., Yan T. (2023). Three cases of obinutuzumab treatment for rituximab-resistant phospholipase A2 receptor-associated membranous nephropathy. Chin. J. Nephrol..

[67] Zhu S., Chen D., Xu F., Li Z., Tu Y. (2026). A Decade-Long Journey of Steroid-Dependent IgA Nephropathy with Minimal Change Disease from Immunosuppressants to Rituximab and then to Obinutuzumab: A Case Report. Case Rep. Nephrol. Dial..

[68] Teisseyre M., Cremoni M., Boyer-Suavet S., Ruetsch C., Graça D., Esnault V.L.M., Brglez V., Seitz-Polski B. (2022). Advances in the Management of Primary Membranous Nephropathy and Rituximab-Refractory Membranous Nephropathy. Front. Immunol..

[69] Zhao N., Chen P., Chen J., Wang B., Tang X., Tang L., Wang Z., Zhang Y. (2026). Obinutuzumab is effective for refractory primary membranous nephropathy. BMC Nephrol..

[70] Jin Y., Huang Y., He X. (2026). Obinutuzumab-induced acute thrombocytopenia and leukopenia in ANCA-associated glomerulonephritis: Case report and literature review. Front. Immunol..

[71] Chen J., Li D.T., Feng X., Zhang B.R. (2025). Case Report: Subcutaneous ofatumumab for patients with immunosuppressant-dependent or ineffective primary membranous nephropathy. Front. Immunol..

[72] Zhang Y., Guo M., Bian Q., Li S., Sun J., Qin Z., Xu J., Dong R. (2026). Comparison of the efficacy of obinutuzumab versus rituximab combined with tacrolimus in the treatment of refractory membranous nephropathy. BMC Nephrol..

[73] Sessa C., Galeano D., Zanoli L., Delsante M., Rossi G.M., Morale W. (2025). Obinutuzumab in membranous nephropathy: A potential game-changer in treatment. Drugs Context.

[74] Beers S.A., French R.R., Chan H.T.C., Lim S.H., Jarrett T.C., Vidal R.M., Wijayaweera S.S., Dixon S.V., Kim H., Cox K.L. (2010). Antigenic modulation limits the efficacy of anti-CD20 antibodies: Implications for antibody selection. Blood.

[75] Lim S.H., Vaughan A.T., Ashton-Key M., Williams E.L., Dixon S.V., Chan H.T.C., Beers S.A., French R.R., Cox K.L., Davies A.J. (2011). Fc gamma receptor IIb on target B cells promotes rituximab internalization and reduces clinical efficacy. Blood.

