# Supplementary material for: Switching Beyond Rituximab in Primary Glomerular Diseases: A Systematic Review of Within-Patient Efficacy and Safety of Non-Rituximab Anti-CD20 Antibodies
Source: Antibodies (Basel). 2026 Oct 10;15(5):95. doi: 10.3390/antib15050095 (PMC13700698; doi:10.3390/antib15050095)
Supplement: Supplementary file 1 [file antibodies-15-00095-s001.zip › antibodies-4513250-supplementary.pdf]

Table S1. Patient-level and eligible-subgroup clinical and laboratory outcomes in membranous nephropathy.

Study-ID / Patient-ID	Follow-up (months)	Baseline Kidney function	Baseline proteinuria	Baseline albumin (g/L)	Baseline B cells (cells/uL)	Baseline anti-PLA2R Ab (RU/mL)	Baseline anti-Ritu Ab	End-FU Kidney function	End-FU proteinuria	End-FU albumin (g/L)	End-FU B cells (cells/µL)	end-FU Anti-PLA2R Ab (RU/mL)	End F-U anti-RTX Ab	Time to response	CR	PR	Relapse	Note
Boyer-Suavet.Sonia.2020.FI #1	3	NR^	*6g/g	NR	NR	*30	23 ng/mL	NR	0.4 g/g	NR	NR	negative	NR	3	no	yes	no	^only aggregate data in Anti-RTX Ab (n.10): S-creat 127 µmol/L (IQR 105-149) **High risk of duplication with Teisseyre.2025.KIR**
Boyer-Suavet.Sonia.2020.FI #2	3	NR^	*7 g/g	NR	NR	*40	450 ng/mL	NR	2 g/g	NR	NR	negative	440 ng/mL at month-3	3	no	yes	no	
Boyer-Suavet.Sonia.2020.FI #3	3	NR^	NR	NR	NR	NR	Positive	NR	NR	NR	NR	negative	NR	3	unknown	yes	no	
Cheng.Xueyang.2025.FI #1	9	NR^	3.95 g/day	NR	NR^	NR	87.6 ng/mL	NR	0.29 g/day	NR	NR	NR	NR	3	yes	N.A.	no	^only aggregate data in previous RTX (n.29): eGFR: 81(IQR 61-96),
Cheng.Xueyang.2025.FI #2	6	NR^	7.7 g/day	NR	NR^	NR	122.99 ng/mL	NR	2.3 g/day	NR	NR	NR	NR	3	no	yes	no	
Cheng.Xueyang.2025.FI #3	6	NR^	6.24 g/day	NR	NR^	NR	5.88 ng/mL	NR	1.66 g/day	NR	NR	NR	NR	3	no	yes	no	

Cheng.Xueyang.2025.FI #4	9	NR^	6.21 g/day	NR	NR^	NR	2.44 ng/mL	NR	1.27 g/day	NR	NR	NR	NR	3	no	yes	no	Albumin: 26 (IQR 20-47)
Chen.Zewei.2025.CKJ #1	20	eGFR 62.1 mL/min/1.73 m²	8.15 g/day	25.4	78	21.77	NR	eGFR 70 mL/min/1.73 m²	1.98 g/day	39	NR	<2	NR	8	no	yes	no	
Chen.Zewei.2025.CKJ #2	8	eGFR 84.1 mL/min/1.73 m²	6.02 g/day	17.3	18	234.29	NR	eGFR 89.9 mL/min/1.73 m²	2.14 g/day	33.8	NR	<2	NR	2.5	no	yes	no	
Chen.Zewei.2025.CKJ #3	18	eGFR 44.5 mL/min/1.73 m²	8.99 g/day	16.9	3	141.35	NR	eGFR 42.7 mL/min/1.73 m²	0.21 g/day	43.2	NR	<2	NR	9	yes	no	no	
Chen.Zewei.2025.CKJ #4	24	eGFR 49.5 mL/min/1.73 m²	14.6 g/day	22.6	0	86.15	NR	eGFR 58.9 mL/min/1.73 m²	0.73 g/day	44.8	NR	<2	NR	10	no	yes	no	
Chen.Zewei.2025.CKJ #5	6	eGFR 73.9 mL/min/1.73 m²	20.4 g/day	20.4	19	100.91	NR	eGFR 119.2 mL/min/1.73 m²	8.25 g/day	21.7	NR	5.18	NR	never	no	no	N.A.	Proteinuria decreased
Chen.Zewei.2025.CKJ #6	6	eGFR 34.5 mL/min/1.73 m²	27.8 g/day	24.3	4	2.66	NR	eGFR 52.1 mL/min/1.73 m²	3.43 g/day	35.8	NR	<2	NR	6	no	yes	no	
Chen.Zewei.2025.CKJ #7	7	eGFR 43.6 mL/min/1.73 m²	5.4 g/day	28.9	9	<2	NR	eGFR 45.6 mL/min/1.73 m²	0.16 g/day	45.5	NR	<2	NR	1	yes	no	no	
deLaPradaAlvarez.FranciscoJose.2024.Nefrologia	9	eGFR 42 mL/m	5.6 g/g	20	NR	109.44	NR	eGFR 50 mL/m	1.5 g/g	31	NR	Negative	NR	6	no	yes	no	

		in/1.73 m^2						in/1.73 m^2										
Guo.Jianying.2026.FI	18	S.Creat at 72.5 μmol/L	7.8 g/g	25.3	0%	8.95	36.83 UI/mL	eGFR 62 mL/min/1.73 m^2	0.51 g/g	43.5	7.23%	1.88	NR	8	no	yes	no	
Hudson.Rebecca.2022.BMCN #1	15	eGFR 20 mL/min/1.73 m^2	21 g/day	<15	10	279	NR	eGFR 72 mL/min/1.73 m^2	7.56 g/day	42	20	<2RU /mL	NR	never	no	no	N. A.	>50% reduction, normalization of albumin and improved renal function
Hudson.Rebecca.2022.BMCN #2	12	eGFR >90 mL/min/1.73 m^2	5.08 g/day	24	NR	55	NR	eGFR >90 mL/min/1.73 m^2	1.43 g/day	36	30	<2.1	NR	9	no	yes	no	
Jha.VijoyKumar.2025.IJN #1	10	eGFR 75 mL/min/1.73 m^2	17.89 g/g	18	NR	293	NR	eGFR 68 mL/min/1.73 m^2	10.8 g/g	28	<5	132	NR	never	no	no	N. A.	40% reduction of Proteinuria
Jha.VijoyKumar.2025.IJN #2	11	eGFR 108 mL/min/1.73 m^2	around 3.5 g/day	26	NR	NR	NR	eGFR 95 mL/min/1.73 m^2	230 mg/g	41	<5	negative	NR	3	yes	N.A.	no	
Jha.VijoyKumar.2025.IJN #3	10	eGFR 48 mL/min/1.73 m^2	30.72 g/day	20	NR	369.01	NR	eGFR 46 mL/min/1.73 m^2	540 mg/g	36	<5	NR	NR	3	no	yes	no	
Jha.VijoyKumar.2025.IJN #4	10	eGFR 91 mL/min/1.73 m^2	3.8 g/day	24	NR	77.7	NR	eGFR 82 mL/min/1.73 m^2	242 mg/g	34	<5	14	NR	3	yes	N.A.	no	
Jha.VijoyKumar.2025.IJN #5	13	eGFR 122 mL/m	9 g/g	14	NR	246	NR	eGFR 122 mL/m	220 mg/g	32	<5	negative	NR	6	yes	N.A.	no	

		in/1.73 m^2						in/1.73 m^2										
Jha.VijoyKumar.2025.IJN #6	18	eGFR 81 mL/min/1.73 m^2	10 g/day	NR	NR	342	NR	eGRR 123 mL/min/1.73 m^2	150 mg/g	39	<5	negative	NR	6	yes	N.A.	no	
Klomjit.Nattawat.2020.AJKD #1	18	eGFR 35 mL/min/1.73 m^2	6.8 g/day	25	0	92	NR	eGFR 49 mL/min/1.73 m^2	1.1g/d	43	19	5	NR	12	no	yes	no	
Klomjit.Nattawat.2020.AJKD #2	9	eGFR 40 mL/min/1.73 m^2	20.4 g/day	28	NR	125	NR	eGFR 57 mL/min/1.73 m^2	6.8 g/day	38	0	<2	NR	never	no	no	N.A.	50% reduction of proteinuria
Klomjit.Nattawat.2020.AJKD #3	24	eGFR 47 mL/min/1.73 m^2	19.5 g/day	25	49	12	NR	eGFR 47 mL/min/1.73 m^2	1.5 g/day	41	396	<2	NR	18	no	yes	no	
Li.Xue-Qi.2025.DDDT#1	7		20.4 g/day	NR	44.85	37.97	NR	NR	3.6 g/day	NR	0	3.52	NR	1	no	yes	N.A.	82.3 % reduction of proteinuria at month1 after the last OBI infusion
Li.Xue-Qi.2025.DDDT#2	NR		10.08 g/day	NR	0	461.83	NR	NR	5.38 g/day	NR	3	57.4	NR	NR	no	yes	N.A.	50 % reduction of proteinuria at month1 after the last OBI infusion

Liu.Yang.2026.KIR#1	9	S-creat 163 μmol/L	12 g/d	29.1	NR	31.58	145.39 IU/ml	S-creat 161.9 μmol/L	1.64 g/d	45.9	NR	<5	NR	NR	no	yes	no	
Liu.Yang.2026.KIR#2	10	S-creat 87.6μmol/L	3.86 g/d	39.9	NR	21.37	11.6 IU/ml	S-creat 88.9 μmol/L	0.46	47.7	NR	<5	NR	NR	no	yes	no	
Liu.Yang.2026.KIR#3	10	S-creat 74.5 μmol/L	7.1 g/d	27.7	NR	30.56	488.76 IU7ml	S-creat 63.8μmol/L	1.28	41.2	NR	<5	NR	NR	no	yes	no	
Liu.Yang.2026.KIR#4	11	S-creat 152.4 μmol/L	4.39 g/d	26.6	NR	105.34		S-creat 109 μmol/L	5.75	28.5	NR	3.18	NR	11	no	yes	yes	
Liu.Yang.2026.KIR#5	6	S-creat 71.7 μmol/L	3 g/d	35.2	NR	7,99	>1500 IU/ml	S-creat 73.3 μmol/L	1.1 g/d	40.8	NR	<5	NR	NR	No	yes	no	Short F-U

Liu.Yang.2026.KIR#6	6	S-creat 74.6 µmol/L	3.1 g/d	25.6	NR	<5	27.5 IU/ml	S-creat 69.5 µmol/L	0.4	41.3	NR	<5	NR	NR	No	yes	no	Short F-U
Lin.Yuxin.2024.KM#1-12	^13.6 (IQR 11.9-14.9)	^ S-creat 86.2 ±21.8 µmol/L	^ 3.5 g/g (IQR 1.9-7.1)	^ 27.2 ± 8.2	^72.0 (IQR 10.0-227.5).	^ 16.5 (IQR 6.1-170.3)	NR	stable	°0.6 g/g (IQR 0.1-1.6)	°43.8 ± 2.9	NR	NR	NR	NR	4/12 (33.3 %)	8/12 (66.7 %)	unclear	^aggregate of whole cohort °aggerate of whole cohort at 12 months 1 of full-cohort responders relapsed 9 months after achieving PR
Naik.Sachin.2023.KIR #1	18	eGFR 27 mL/min/1.73 m²	12.03 g/day	25.5	NR	200.71	NR	eGFR 43 mL/min/1.73 m²	2.05	39.9	<5	2.55	NR	NR	no	yes	no	
Naik.Sachin.2023.KIR #2	10	eGFR 14 mL/min/1.73 m²	9.71 g/day	27	NR	125.1	NR	eGFR 30 mL/min/1.73 m²	3.39 g/day	41.3	<5	0.6	NR	NR	no	yes	no	
Sethi.Supreet.2020.KIR #1	.NR	S-Creat 1.1 mg/dL	14.38 g/g	29	NR	negative	NR	* 1.1 mg/dL	* 0.3 g/day	* 39	NR	NR	NR	* 6	yes	N.A.	no	One patient had secondary MN

Sethi.Supreet.2020.KIR #2	NR	S-Creat 2.1 mg/dL	11.3 g/g	17	NR	633	NR	* 1.7 mg/dL	* 6 g/day	* 20	NR	5.1	NR	* 6	no	no	N.A.	but he is undetectable. Overall cohort median follow-up: 18 months (IQR 9-24)
Sethi.Supreet.2020.KIR #3	NR	S-Creat 0.9 mg/dL	10.73 g/g	27	NR	39	NR	* 0.9 mg/dL	* 2.5 g/day	* 38	NR	1.8	NR	* 6	no	yes	no	
Sethi.Supreet.2020.KIR #4	NR.	S-Creat 1.3 mg/dL	7.8 g/g	21	NR	261	NR	* 1.2 mg/dL	* 0.3 g/day	* 39	NR	NR	NR	* 6	yes	N.A.	no	
Sethi.Supreet.2020.KIR #5	NR	S-Creat 0.9 mg/dL	4.5 g/g	39	NR	negative	NR	* 1 mg/dL	* 0.3 g/day	* 43	NR	NR	NR	* 6	yes	N.A.	no	
Sethi.Supreet.2020.KIR #6	NR	S-Creat 1.1mg/dL	3.6 g/g	25	NR	negative	NR	* 1 mg/dL	* 0.3 g/day	* 34	NR	NR	NR	* 6	yes	N.A.	no	
Shi.Guo-Qing.2025.NDT#1	18	NR	NR	NR	NR	NR	NR	NR	NR	NR	NR	NR	NR	1	yes	N.A.	no	
Shi.Guo-Qing.2025.NDT#2	12	NR	NR	NR	NR	NR	NR	NR	NR	NR	NR	NR	NR	3	yes	N.A.	no	
Shi.Guo-Qing.2025.NDT#3	6	NR	NR	NR	NR	NR	NR	NR	NR	NR	NR	NR	NR	4	No	yes	no	Values reported as aggregate
Shi.Guo-Qing.2025.NDT#4	7	NR	NR	NR	NR	NR	NR	NR	NR	NR	NR	NR	NR	8	No	Yes	no	
Shi.Guo-Qing.2025.NDT#5	6	NR	NR	NR	NR	NR	NR	NR	NR	NR	NR	NR	NR	3	yes	N.A.	no	
Sridharan.Krita.2025.JNephrol #1	13	S-Creat	NR	NR	NR	NR	NR	NR	NR	NR	NR	NR	NR	6	yes	N.A.	no	

		52 µmol/L																
Sridharan.Krita.2025.JNephrol #2	9	S-Creat 133 µmol/L	1,700 mg/mmol	29	NR	19	NR	S-Creat 158 µmol/L	10 mg/mmol	44	<0.1%	negative	NR	9	yes	N.A.	no	
Sridharan.Krita.2025.JNephrol #3	4	S-Creat 60 µmol/L	438 mg/mmol	32	NR	50	NR	S-Creat 74µmol/L	6.7mg/mmol	40	<0.1%	negative	NR	1	yes	N.A.	no	
Teisseyre.Maxime.2022.KIR	12	NR	6.5 g/day	NR	96	427	>100 ng/mL	NR	2.2 g/day	NR	1	0	>100 ng/mL	6	no	yes	no	**High risk of duplication with Teisseyre.2021.KIR**
Wang.Jinmeng.2025.FI #1	12	S-Creat 119 µmol/L	11.17 g/day	27	1.79	52.8	NR	S-Creat 76 µmol/L	9.45 g/day	29.2	4.41	14.9	NR	4	no	yes	yes at month 10	
Wang.Jinmeng.2025.FI #2	12	S-Creat 73 µmol/L	13.85	29.5	1.4	negative	NR	S-Creat 73. µmol/L	4.45 g/day	39.5	0.04	NR	NR,	12	yes	N.A.	no	
Wang.Linna.2026.KM#1	9	^ S-Creat 99.4 ±42.33 µmol/L	4.2 g/d	27.7	NR	26.85	NR	°S-Creat 8.54 ±57.08 µmol/L	0.16 g/d	37.29	NR	<2	NR	NR	yes	N.A	no	^ Aggregate results for second-line therapy ° Aggregate results for second-
Wang.Linna.2026.KM#2	3	^ S-Creat 99.4 ±42.33 µmol/L	9.16 g/d	21.77	^60.5 (IQR 51-28.5)	67.23	NR	°S-Creat 8.54 ±57.08 µmol/L	1.86 g/d	38	°2 (IQR 2-15.5)	11.23	NR	NR	no	Yes	no	

Wang.Linna.2026.KM#3	9	^ S-Creat 99.4 ±42.33 μmol/L	3.63 g/d	36.67	^60.5 (IQR 51-28.5)	<2	NR	°S-Creat 8.54 ±57.08 μmol/L	1.61 g/d	39.76	°2 (IQR 2-15.5)	<2	NR	NR	no	yes	no	line therapy on 6 months
Wang.Linna.2026.KM#4	6	^ S-Creat 99.4 ±42.33 μmol/L	4.62 g/d		^60.5 (IQR 51-28.5)	10.12	NR	°S-Creat 8.54 ±57.08 μmol/L	1.67 g/d	35.24	°2 (IQR 2-15.5)	<2	NR	NR	no	yes	no	
Wang.Linna.2026.KM#5	14	^ S-Creat 99.4 ±42.33 μmol/L	12.1 g/d	17.17	^60.5 (IQR 51-28.5)	49.86	NR	°S-Creat 8.54 ±57.08 μmol/L	0.83 g/d	38.77	°2 (IQR 2-15.5)	<2	NR	NR	no	yes	no	
Wang.Linna.2026.KM#6	14	^ S-Creat 99.4 ±42.33 μmol/L	6.81 g/d	30.12	^60.5 (IQR 51-28.5)	293.45	NR	°S-Creat 8.54 ±57.08 μmol/L	2.76 g/d	33.49	°2 (IQR 2-15.5)	2.98	NR	NR	no	yes	no	
Wang.Linna.2026.KM#7	6	^ S-Creat 99.4 ±42.33 μmol/L	4.42 g/d	32.6	^60.5 (IQR 51-28.5)	<2	NR	°S-Creat 8.54 ±57.08 μmol/L	1.07	39.02	°2 (IQR 2-15.5)	<2	NR	NR	no	yes	no	
Wang.Linna.2026.KM#8	10	^ S-Creat 99.4 ±42.33 μmol/L	5.08 g/d	31.47	^60.5 (IQR 51-28.5)	19.19	NR	°S-Creat 8.54 ±57.08 μmol/L	3.75	36.22	°2 (IQR 2-15.5)	3.1	NR	NR	no	no	N. A.	
Wang.Linna.2026.KM#9	6	^ S-Creat 99.4 ±42.33 μmol/	5.76	10.4	^60.5 (IQR 51-28.5)	99.83	NR	°S-Creat 98.54 ±57.08 μmol/	16.33	14.16	°2 (IQR 2-15.5)	<2	NR	NR	no	no	N. A.	

		L µmol/ L						L µmol/ L									
Xu.Qiuyu.2024.CJIM	5	S-Creat 213 µmol/ L	18.5 g/day	33	13	negative	NR	S-Creat 162 µmol/ L/l	<3.5 g/g	36	negative	negative	NR	never	no	no	N. A.	proteinuria reduction > 50% and normalization of serum albumin
Zhang.Yao.2024.FrontPharmacol	12	*S-Creat 159-160 µmol/ L	15 g/day	16.2	0	84	NR	*S-Creat 144 µmol/ L	12 g/day	* 33 g/L	NR	negative	NR	never	no	no	N. A.	proteinuria reduction > 50% and normalization of serum albumin
Zhao.Xiangling.2026. FrontMed#1-9	Likely 12	NR	NR	NR	NR	NR	NR	NR	NR	NR	NR	negative	NR	NR	NR	NR	NR	7/9 had CR or PR
Zheng.Zhenfeng.2023.ChinJNephrol #1	10	S-Creat 71 µmol/ L	13.23 g/day	13	NR	270.47	NR	NR	2.175 g/day	38	NR	negative	NR	7	no	yes	no	
Zheng.Zhenfeng.2023.ChinJNephrol #2	7	S-Creat 48 µmol/ L	9.31 g/day	15	NR	44.36	NR	NR	1.890 g/day	NR	NR	negative	NR	10	no	yes	no	
Zheng.Zhenfeng.2023.ChinJNephrol #3	8	S-Creat 106	13.56 g/d	20	NR	5.5	NR	NR	1.92g/ day	NR	NR	negative	NR	10	no	yes	no	

		μmol/L																
Zamora.JorgeIvan.2025.Nefrologia#1	3	S-Creat 1 mg/dL	7.8 g/g	26	170	NR	NR	0.92	9.6 g/g	24	NR	NR		unknown	no	no	N.A.	Lost at F-U at month3

Footnotes: * extrapolated from graph of the report, NR not reported, S-Creat Creatinine, MN membranous nephropathy.

Table S2. Aggregate clinical and laboratory outcomes in membranous nephropathy cohort studies.

Study-ID	Follow-up (months)	Baseline kidney function	Baseline proteinuria	Baseline albumin g/L	Baseline B cells (cells/uL)	Baseline anti-PLA2R Ab RU·mL⁻¹	Baseline anti-Ritu Ab	End F-U kidney function	End-FU proteinuria	End-FU albumin (g/L)	End-FU B cells (cells/µL)	end-FU Anti-PLA2R Ab	End F-U anti-RTX Ab	Time to response	CR (%)	PR (%)	Relapse	Note
Cheng.Xi.2025.FI	10.0 (IQR 6.8-13.0)^	eGFR 66.1 mL/min/1.73 m² (IQR 44.3-107.3)^	5.0 (IQR 3.6-10.3)^ g/day.	26.0 (IQR 22.0-31.0)^	109.0 (IQR 34.3-266.0)^	9.3 (IQR 5.0-51.0)^	NR	eGFr 78.2 mL/min/1.73 m² (IQR 48.6-100.5)^	0.9 (IQR 0.3-2.3)^ g/day.	42.0 (IQR 39.0-44.0)^	2.0 (IQR 1.0-3.0)^	NR	NR	10 (IQR 6.8-13)	46.7	40	6.70	^ Reported only for alternative therapy (40pts)
Dong.Rui.2025.AJNMU	12	eGFR 82 (IQR 70-100)^ mL/min/1.73 m²	5.6 (IQR 2.9-9.2)^ g/day	24 (IQR 20-32)^ g/L	NR	112.5 (IQR 66.4-242.7)^	NR	eGFR 83.0 (IQR 70.5-91.0)^ mL/min/1.73 m²	0.5 (IQR 0.1-1.2)^ g/day	44.0 (IQR 42.3-45.5)^ g/L	NR	2 (IQR 2-3.5)^ RU·mL⁻¹	NR	unclear	18.2	81.8	NR	Clinical remission outcomes are reported for the 11 RTX-pretreated patients; ^laboratory and immunological values refer to the full cohort and could not be separated for the RTX-pretreated subgroup.
Li.Huxian.2026.AJN	12.6 +/- 5.0	eGFR <60 mL/min/1.73 m²	8.7 +/- 3.1 g/day	22.6 +/- 3.6	NR	28.3 (IQR 5.9-135.7);	NR	eGFR improved or stabilized	2.4 +/- 2.1 g/day	35.4 +/- 5.2	NR, but decreased	de-creased by medians of 94.6%	NR	4.4 (IQR 4.0-6.0)	52.9	35.4	no	Urinary protein decreased by 75.1%
Li.Luyao.2025.IUM	^Responder (n.21) 12 (IQR12-14) Non -eposn	^eGFR 95±31.23 mL/min/1.73 m²	^ 7.92± 4.99 g/g	^30.08±8.36	NR	^55.50 (IQR 11.55–129.90)	NR	^Responder 100.73 ± 26.80 mL/min/1.73 m² Non - respond	^Responder 1(0.62-2.06) g/g Non - responder 4.65 (3.14-	^Responder 42.34 ± 4.81 Non - responder	NR	NR	NR	9	3	16	no	^ Results only aggregate<br>21 PLA2R positive before obinutuzumab, after 2 PLA2R

	der (n 6) 12(IQR 12-18)							er 82.62 ± 40.76 mL/min/1.73 m²	17.61) g/g	34.80 ± 8.24								positive after obinutuzumab
Podesta.ManuelAlfredo.2024.AJKD	23.9 (IQR 17.7-24.0).	RTX -Resistant: 41.9 (37.4-67.7); RTX-intolerant: 67.4 (48.5-89.9)	RTX -Resistant:: 10.0 +/- 4.7 g/day; RTX-intolerant: 7.7 +/- 3.5 g/day.	RTX -Resistant: 25 +/- 5 ; RTX-intolerant: 26 +/- 6 g/	RTX -Resistant: 163 +/- 97; RTX-intolerant: 128 +/- 69	RTX -Resistant: 66 (18-308); RTX-intolerant: 43 (10-83)	NR	Increased in whole cohort approximately by 17%, in RTX-intolerant achieved higher values	*70%reduction in RTX-intolerant 50% reduction in RTX -Resistant with significant difference at month 12	*50% reduction in RTX-resistant and RTX-intolerant	in whole cohort *250	in whole cohort *90% reduction	NR	5	Yes+°	Yes°		°CR or PR in 3/10 RTX -Resistant and 7/7 RTX-intolerant. **High risk of duplication with Teisseyre.2025.KIR**
Su.Wenyan.2025.CKJ	9.0 (IQR 6.75-12.0)	creatinine 66.85 μmol/L (IQR 49.65-85.93)	5.49 g/day (IQR 3.56-10.80)	27.15 g/L (IQR 22.00-29.33)	48.56 (IQR 24.49-182.6)	29.81 (IQR 4.57-68.59)	NR	creatinine 67.45 (IQR 52.55-85.40)	1.46 (0.91-3.04)	39.70 (36.73-42.20)	20 (100%) achieved CD19+ depletion	0.00 (0.00-5.59)	NR	4.50 (IQR 3.00-8.25	10	70	no	short F-U
Su.Xiaole.2024.KIR	9.4 months (IQR 7.8-11.3	Mean eGFR 83.8 +/- 30.2 mL/min/1.73 m2 in RTX-resistant	Mean 24-hour urinary protein 4.5 +/- 2.4 g/day	Mean serum albumin 33.3 +/- 7.3 g/L in the RTX-resistant	Median CD19+ count 92.9 cells/μL (IQR 55.2-198.0)	NR	NR	no significant change in eGFR	ranged from 3.1 to 6.7 g/day.	Among the five rituximab-resistant nonresponders 33.2 to 38.0 g/L	<5 cells/μL	anti-PLA2R <14 RU/mL, occurred in 7/10 (70%) evaluable positive patients	NR		21.4	42.9	no	short F-U

Teisseyre.Maxime.2025.KIR	Median follow-up: 96 weeks (IQR 52-124)	eGFR 59 mL/min/1.73 m² (29-83)	5.9 g/g (4.7-7.5)g/g	25.2 g/L (22.4-30.0)	NR	eGFR 50 mL/min/1.73 m² (IQR 27-217)	28 ng/mL (12-55)	stable	NR	NR	13/14 (93%) negative	12/13 (92%) negative	Anti-RTX Ab positivity in 4/9 (44%)	6	83% OBI	100% in OFA	33%	
Tian.Li.2026.Open Medicine	9 months (range 6-30)	S-Creat 81.1 ± 34.1 µmol/L	11.3 ± 5.6 g/day	21.7 ± 5.5	111 ± 84	S-Creat 133.85 (range 10.88-820.41) µmol/L	NR	NR	NR	NR	NR	13/21 (62%) negative	NR	14	16	64	8%	80% had CR or PR
Trillini.Matias.2026.KIR	At least 12 (group A), 10 pts (50%) 24 (groupB)	S-creat 61.42 ± 13.92 µmol/L	Group A: 5.70 g/day (IQR 4.70-8.15); Group B: 6.65 g/day (IQR 4.90-8.50)	Group A: 29 ± 6.5; Group B: 2.76 ± 0.75	Group A: 187.85 ± 130.71 Group B: 2.76 ± 0.75	Group A: S-Creat107 (IQR 81-284) Group B: S-Creat 250.50 (59.00-398.50) µmol/L	NR	Group A: 67.45 ± 13.37, Group B 66.15 ± 17.15	Group A: 1.35 g/day (IQR 0.55-2.75; P<0.001). Group B: 1.95 (1.50-2.30) g/day	Group A: 39.2 ± 2.9 Group B: 4.10 ± 0.32 g/dL	Group A: 50.30 ± 67.14. 24 Group B: 296.40 ± 213.30	Group A: 1.90 RU/mL (IQR 1.90-1.99) Group B: 1.90 (1.90-1.95) RU/mL	NR	12	20	60	no	Prospective, single-center, single-arm, open-label pilot interventional study

Footnotes: NR not reported, RTX rituximab, the descriptive value was reported as median (IQR) or as mean ± SD according to the study report.

Table S3. Patient-level clinical and laboratory outcomes in minimal change disease.

Study-ID / Patient-ID	Follow-up	Baseline Kidney function	Baseline proteinuria	Baseline albumin (g/L)	Baseline B cells (cells/µL)	End-FU kidney function	End-FU proteinuria	End-FU albumin (g/L)	End-FU B cells (cells/µL)	Time to response	CR	PR	Relapse	Note
Angeletti.Andrea.2025.KIR	12	eGFR 115 ml/min/1,73m^2	6.3 g/d	21	NR	stable	* 2.7 g/d	*32	NR	9	no	yes	no	
Chen.Zewei.2025.CKJ #8	7	eGFR 162.5 ml/min/1,73m^2	1.49 g/day	35.8	3	eGFR 162.5	0.03 g/day	44	NR	1	yes	N.A.	no	
Chen.Zewei.2025.CKJ #9	12	eGFR 100.2 ml/min/1,73m^2	3.23	38.2	154	eGFR 118.3	3.62 g/day	21.8	NR	1	yes	N.A.	yes at 12 months	
Chen.Zewei.2025.CKJ #10	6	eGFR 125.5 ml/min/1,73m^2	3.84	33	276	eGFR 129.5	0.06 g/day	42.5	NR	1	yes	N.A.	no	
Gauci.Pierre-Alexis.2025.RF	25	* eGFR 120 ml/min/1,73m^2	NR	*41	NR	* eGFR 120	Minimal	*42	NR	12	yes	N.A.	no	pregnacy
Jin.Li.2024.NDT	9.9	eGFR 145 ml/min/1,73m^2	1.1	33.2	1.57	stable	*Unremarkable	* 40	0	1	yes	N.A.	no	
Lin.Yuxin.2025.AJN#1	25	eGFR 110.6±18.0^ ml/min/1,73m^2	2.3 (0.1, 6.2) ^ g/g	36.4 (22.8, 42.4)^	69.5 (2.0, 333.8)^	NR	NR	NR	NR	NR	yes	N.A.	no	^ whole cohort reported as mean ± SD
Lin.Yuxin.2025.AJN#2	20	eGFR 110.6±18.0^	2.3 (0.1, 6.2) g/g	36.4 (22.8, 42.4)	69.5 (2.0, 333.8)	NR	NR	NR	NR	NR	yes	N.A.	no	^ whole cohort reported as mean ± SD
Lin.Yuxin.2025.AJN#3	17	eGFR 110.6±18.0^ ml/min/1,73m^2	2.3 (0.1, 6.2)^ g/g	36.4 (22.8, 42.4)^	69.5 (2.0, 333.8)^	NR	NR	NR	NR	NR	yes	N.A.	no	^ whole cohort reported as mean ± SD

Lin.Yuxin.2025.AJN#4	12	eGFR 110.6±18.0^ ml/min/1,73m²	2.3 (0.1, 6.2) ^g/g	36.4 (22.8, 42.4)^	69.5 (2.0, 333.8)^	NR	NR	NR	NR	NR	yes	N.A.	no	^ whole cohort reported as mean ± SD
Lin.Yuxin.2025.AJN#5	12	eGFR 110.6±18.0^ ml/min/1,73m²	2.3 (0.1, 6.2)^ g/g	36.4 (22.8, 42.4)^	69.5 (2.0, 333.8)^	NR	NR	NR	NR	NR	yes	N.A.	no	^ whole cohort reported as mean ± SD
Lin.Yuxin.2025.AJN#6	15	eGFR 110.6±18.0^ ml/min/1,73m²	2.3 (0.1, 6.2) g/g^	36.4 (22.8, 42.4)^	69.5 (2.0, 333.8)^	NR	NR	NR	NR	NR	yes	N.A.	no	^ whole cohort reported as mean ± SD
Lin.Yuxin.2025.AJN#7	12	eGFR 110.6±18.0^ ml/min/1,73m²	2.3 (0.1, 6.2)^ g/g	36.4 (22.8, 42.4)^	69.5 (2.0, 333.8)^	NR	NR	NR	NR	NR	yes	N.A.	no	^ whole cohort reported as mean ± SD
Lin.Yuxin.2025.AJN#8	12	eGFR 110.6±18.0^ ml/min/1,73m²	2.3 (0.1, 6.2)^ g/g	36.4 (22.8, 42.4)^	69.5 (2.0, 333.8)^	NR	NR	NR	NR	NR	yes	N.A.	no	^ whole cohort reported as mean ± SD
Loeffler.Thomas.2025.Kidney Med	36	*S-Creat 50-70 µmol/L	NR	* 25-30 g/L	NR	stable	absent	* 44	NR, in normal range	* 1	yes	N.A.	no	
Wang.Qiang.2024.FI	12	Normal	11.38 g/g	18-20	64	stable	absent	46	80-90	2	yes	N.A.	no	
Zamora.JorgeIvan. 2025.Nefrologia#2	12	S-Creat 5.81 mg/dL	24 g/g	20	0	S-Creat 1.21mg/dL	2.9 g/g	41	0	3	no	yes	no	
Zamora.JorgeIvan.2025.Nefrologia#3	12	S-Creat 1.46 mg/dL	3	28	100	S-Creat 1.65 mg/dL	1.04 g/g	35	40	3	no	yes	no	

Footnotes: * extrapolated from graph of the report, NR not reported, N.A. not applicable, S-Creat Creatinine

Table S4. Patient-level clinical and laboratory outcomes in focal segmental glomerulosclerosis.

Study-ID / Patient-ID	Follow-up (months)	Baseline Kidney function	Baseline proteinuria	Baseline albumin (g/L)	Baseline B cells (cells/µL)	End-FU Kidney function	End-FU proteinuria	End-FU [illegible]	End-FU B cells (cells/µL)	Time to response	CR	PR	Relapse	Note
ChiliquingaMorales.Byron.2025.Nefrologia	11	Normal	6.5 g/day	21	NR	Normal	4.4 g/day	29	NR	N.A.	no	no	N.A.	
Henriques.AndreiaRita.2026.FN	11.5	109.9 ml/min/1,73m²	1.1 g/day	34	12.5	105.7	0.2 g/day	38	0	2	yes	N.A.	no	
Lin.Yuxin.2025.AJN#9	26	eGFR 110.6±18.0^ ml/min/1,73m²	2.3 (0.1, 6.2) g/g	36.4 (22.8, 42.4)	69.5 (2.0, 333.8)	NR	NR	NR	NR	NR	Yes, only initially	yes	yes	^ whole cohort. PR->Relapse at month 5->CR
Lin.Yuxin.2025.AJN#10	22	eGFR 110.6±18.0^ ml/min/1,73m²	2.3 (0.1, 6.2)^ g/g	36.4 (22.8, 42.4)^	69.5 (2.0, 333.8)^	NR	NR	NR	NR	NR	yes	N.A.	no	^ whole cohort
Lin.Yuxin.2025.AJN#11	18	eGFR 110.6±18.0^ ml/min/1,73m²	2.3 (IQR 0.1, 6.2)^ g/g	36.4 (IQR 22.8, 42.4)^	69.5 (IQR 2.0, 333.8)^	NR	NR	NR	NR	NR	yes	N.A.	yes	^ whole cohort CR-> relapse at month9 -> CR
Zamora Jorge Ivan.2025.Nefrologia#4	12	S-Creat 0.65mg/dL	11 g/g	25	80	S-Creat 0.66 mg/dL	5.5 g/g	33	140	6	no	yes	yes	

Footnote: NR not reported, N.A. not applicable, S-Creat Creatinine.
